# HIF‐1α‐induced long noncoding RNA LINC02776 promotes drug resistance of ovarian cancer by increasing polyADP‐ribosylation

**DOI:** 10.1002/ctm2.70244

**Published:** 2025-03-21

**Authors:** Yangjun Wu, Yu Zeng, Yong Wu, Xinyu Ha, Zheng Feng, Chaohua Liu, Ziqi Liu, Jiajia Wang, Xingzhu Ju, Shenglin Huang, Linhui Liang, Bin Zheng, Lulu Yang, Jun Wang, Xiaohua Wu, Shengli Li, Hao Wen

**Affiliations:** ^1^ Department of Gynecologic Oncology Fudan University Shanghai Cancer Center, Fudan University Shanghai China; ^2^ Department of Oncology Shanghai Medical College, Fudan University Shanghai China; ^3^ Precision Research Center for Refractory Diseases and Shanghai Key Laboratory of Pancreatic Diseases Shanghai General Hospital, Shanghai Jiao Tong University School of Medicine Shanghai China; ^4^ Key Laboratory of Medical Epigenetics and Metabolism, Institutes of Biomedical Sciences Fudan University Shanghai Cancer Center, Fudan University Shanghai China; ^5^ Accurate International Biotechnology Co. Ltd. Guangzhou China; ^6^ Wuhan Benagen Technology Co., Ltd Wuhan China

**Keywords:** HIF‐1α, LINC02776, ovarian cancer, PARP1, PARPi resistance, platinum resistance

## Abstract

**Background:**

Chemoresistance remains a major hurdle in ovarian cancer (OC) treatment, as many patients eventually develop resistance to platinum‐based chemotherapy and/or PARP inhibitors (PARPi).

**Methods:**

We performed transcriptome‐wide analysis by RNA sequencing (RNA‐seq) data of platinum‐resistant and ‐sensitive OC tissues. We demonstrated the role of LINC02776 in platinum resistance in OC cells, mice models and patient‐derived organoid (PDO) models.

**Results:**

We identify the long noncoding RNA LINC02776 as a critical factor of platinum resistance. Elevated expression of LINC02776 is observed in platinum‐resistant OC and serves as an independent prognostic factor for OC patients. Functionally, silencing LINC02776 reduces proliferation and DNA damage repair in OC cells, thereby enhancing sensitivity to platinum and PARPi in both xenograft mouse models and patient‐derived organoid (PDO) models with acquired chemoresistance. Mechanistically, LINC02776 binds to the catalytic domain of poly (ADP‐ribose) polymerase 1 (PARP1), promoting PARP1‐dependent polyADP‐ribosylation (PARylation) and facilitating homologous recombination (HR) restoration. Additionally, high HIF‐1α expression in platinum‐resistant tissues further stimulates LINC02776 transcription.

**Conclusions:**

Our findings suggest that targeting LINC02776 represents a promising therapeutic strategy for OC patients who have developed resistance to platinum or PARPi.

**Key points:**

- LINC02776 promotes OC cell proliferation by regulating DNA damage and apoptosis signaling pathways.
- LINC02776 binds PARP1 to promote DNA damage‐triggered PARylation in OC cells.
- LINC02776 mediates cisplatin and olaparib resistance in OC cells by enhancing PARP1‐mediated PARylation activity and regulating the PARP1‐mediated HR pathway.
- The high expression of LINC02776 is induced by HIF‐1α in platinum‐resistant OC cells and tissues.

## Graphical abstract

LINC02776 was significantly upregulated in platinum‐resistant ovarian cancer (OC) patients compared to those sensitive to platinum therapy. LINC02776 directly binds catalytic domain of PARP1 to enhance PARP1‐dependent polyADP‐ribosylation, thereby promoting homologous recombination (HR) restoration.

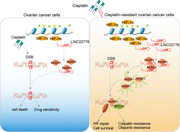

## 1 INTRODUCTION

Based on the global cancer statistics for 2020, it was reported that 313 959 new cases of ovarian cancer (OC) were diagnosed globally, resulting in 207 252 associated fatalities.^1^ High‐grade serous ovarian carcinoma (HGSOC), which is the most common histological variant of epithelial ovarian cancer (EOC), is diagnosed at an advanced stage in 70% of cases, leading to a poor prognosis. The current standard therapeutic approach for this condition typically includes cytoreductive surgery followed by chemotherapy involving platinum‐based drugs; however, the emergence of platinum resistance remains a persistent clinical challenge.^2^ More recently, the widespread adoption of PARP inhibitors (PARPi) for maintenance therapy in advanced OC following a response to first‐line chemotherapy has raised concerns that resistance to both platinum‐based therapies and PARPi may emerge within the next year following treatment.^3^, ^4^ Consequently, elucidating the mechanisms underlying resistance to platinum and/or PARPi, as well as devising strategies to optimise combination therapies, holds significant clinical importance.

Recent studies have identified multiple mechanisms that drive resistance to platinum‐based therapies and PARPi. Notably, PARPi resistance often arises alongside resistance to platinum‐based chemotherapy,^5^ suggesting overlapping pathways. Resistance to cisplatin (DDP), carboplatin or oxaliplatin may result from reduced intracellular drug accumulation, enhanced DNA repair activity or diminished apoptotic signalling pathways.^6^ PARPi resistance can be homologous recombination (HR) dependent or independent. HR‐dependent resistance may arise from the reactivation of BRCA1/2 function^7^, ^8^ or restoration of HR by factors like loss of 53BP1, Shieldin factors, conserved telomere maintenance component 1/DNA polymerase alpha‐primase (CTC1/Polα) or DYNLL1/ATMIN.^9^, ^10^, ^11^ Conversely, HR‐independent resistance mechanisms involve increased drug efflux,^12^ reduced PARP1 trapping,^13^ poly(ADP‐ribose) glycohydrolase (PARG) loss,^14^ and stabilisation of stalled replication forks.^15^ Common elements in resistance to both drug classes include limited intracellular availability of the agent and alterations in DNA repair pathways, such as restored HR and stabilised replication forks. Inadequate drug uptake or increased efflux due to transporter alterations can reduce the intracellular concentration of the therapy, whereas amplified DNA repair activity may result from various pathways, including nucleotide excision repair (NER), nonhomologous end joining (NHEJ), mismatch repair (MMR), base excision repair (BER), and HR.^16^ During the early stage of the DNA damage response, PARP1 is recruited to lesions, and its polyADP‐ribosylation (PARylation) activity is integral to repairing single‐strand breaks (SSB) and double‐strand breaks (DSB), as well as stabilising DNA replication forks.^17^ Despite these insights into resistance mechanisms, further research is needed to overcome chemoresistance and improve survival outcomes for patients with OC.

Long noncoding RNAs (lncRNAs) are a class of RNA molecules exceeding 200 nucleotides (nt) in length and possess limited protein‐coding potential.^18^ Dysregulation of lncRNAs has been implicated in both developmental processes and cancer drug resistance.^19^, ^20^, ^21^ For instance, overexpression of lncMALAT1 promotes autophagy‐mediated cisplatin resistance by upregulating autophagy‐related 12 (ATG12).^22^ Silencing lncANRIL restores drug sensitivity in gastric cancer cells by regulating the multidrug resistance transporters P‐glycoprotein (P‐gp) and multidrug resistance protein 1 (MRP1).^23^ LncCTSLP8 also contributes to cisplatin resistance in OC by enhancing cellular glycolysis.^24^ Although some lncRNAs have been shown to influence DNA damage repair by binding to PARP1, such as LIP (long noncoding RNA interacts with PARP‐1), which modulates BER efficiency,^25^ details of these mechanisms remain incompletely understood. In this study, we establish a link among HIF‐1α, LINC02776, PARP1 and HR restoration in OC. We find that high LINC02776 expression is closely associated with OC resistance to both platinum and PARPi. Furthermore, our results indicate that targeting LINC02776 can suppress tumour growth and modulate chemoresistance in OC cells both in vivo and in vitro.

## 2 MATERIALS AND METHODS

### 2.1 Human specimens

Fresh OC tissue samples were collected from surgical specimens at the Department of Gynecological Oncology, Fudan University Shanghai Cancer Center (FUSCC). The diagnosis of EOC was independently verified by three pathologists. This study received approval from the Medical Ethical Committee of FUSCC (Approval No. 2110244‐9), and all participants gave written informed content following a detailed explanation of the study objectives.

Based on the duration of the platinum‐free interval (PFI) following initial platinum‐based chemotherapy, patients are categorised as either platinum‐sensitive (PFI ≥ 6 months) or platinum‐resistant (PFI < 6 months). Within the FUSCC1 cohort, individuals with platinum‐sensitive recurrent OC had a median PFI of 16.2 months. Conversely, those with platinum‐resistant recurrent OC demonstrated a much shorter median PFI of 3.5 months. The clinical and pathological features of FUSCC1 cohort are provided in Table S1.

### 2.2 RNA sequencing and data analysis

Total RNA was isolated from OC tissues using the Trizol reagent (provided by Life Technologies). Clinical and pathological details of the patients included for RNA‐seq were provided in Table S2. Prior to constructing the library, it is essential to verify the integrity of RNA through denaturing agarose gel electrophoresis and subsequently remove rRNA. The library preparation was conducted using the NEBNext UltraDirectional RNA Library Prep Kit, and its quality was assessed using the Agilent Bioanalyzer 2100. Sequencing was carried out on the Illumina HiSeq 4000 platform. The raw RNA‐seq dataset has been deposited in GEO under accession number GSE214302.

FastQC (version 0.11.9) performs quality assessment on raw RNA‐seq data, and reads with low quality were eliminated by Trimmomatic (version 0.36).^26^ Following this, the processed reads were mapped to the human reference genome (GRCh38) utilising STAR (version 2.5.3a).^27^ Following alignment, StringTie (version 1.2.3)^28^ was used for gene and transcript quantification, utilising GENCODE gene annotation (Release 29)^29^ as the reference. Genes or transcripts with >.1 fragments per kilobase of exon model per million mapped fragments (FPKM) in at least one sample were retained for further analysis. The EdgeR^30^ package was used identify differentially expressed lncRNAs with default settings. LncRNAs meeting the threshold of *p* < .01and |log2(fold change)| > 1 were considered significantly differentially expressed (Table S3).

### 2.3 Gene set enrichment analysis

In the LINC02776 knockdown and control samples, genes were ordered based on their normalised expression levels. These ordered gene lists were then submitted to the GSEA software (version 4.0.3) for further analysis.^31^ For this analysis, fifty hallmark gene sets were obtained from the Molecular Signatures Database (MSigDB).^32^

### 2.4 SiRNA, ASO, shRNA and plasmid transfection and lentiviral transduction

All siRNAs and ASOs (Antisense oligonucleotides) were designed and synthesised by RiboBio (RIBOBIO). For gene knockdown, 100 pmol of siRNA or ASO per well (in a six‐well plate) was mixed with jet‐PRIME transfection reagent (Polyplus) and transfected into OC cells according to the manufacturer's instructions. Short hairpin (shRNA) lentiviruses were used for stable gene knockdown in cells. The shRNA constructs were designed based on the siRNA sequences and ligated into Lenti‐guide‐puro vectors. The human LINC02776 sequence was cloned from A2780 cells and inserted into the PCDH vector to generate PCDH‐LINC02776. The full‐length and truncated open‐reading frame (ORF) sequences of PARP1 were designed based on the functional domain annotations in the UniProt database (https://www.uniprot.org/) cloned into the PCDH‐3 × FLAG vector. Polymerase chain reaction (PCR) primers are listed in Table S4. To produce lentivirus, HEK293T cells were simultaneously transfected with the engineered plasmids along with the packaging plasmid (psPAX2) and envelope plasmid (pMD2.G), which were kindly provided by Dr. Didier. This transfection was performed using the Hieff Trans™ Lipsomal Transfection Reagent (Yeasen Biotechnology) according to the manufacturer's instructions. Viral particles were collected 48 h post‐transfection. The specific sequences for the siRNAs, shRNAs and PCR primers utilised in the cloning process are provided in Table S4.

### 2.5 Culturing patient‐derived organoids (PDOs)

Platinum‐resistant OC ascites were collected from a 49‐year‐old woman (FIGO stage IV) who developed resistance after undergoing platinum‐based adjuvant therapy. Olaparib‐resistant OC tissue samples were obtained from an 80‐year‐old woman (FIGO stage IV) who became resistance to olaparib following 10 months of maintenance therapy. The clinical and pathological features of both patients are provided in detail in Table S5. Tumour assessments were performed at FUSCC using computed tomography (CT) and tumour biomarker analysis. Organoid culture was conducted as described previously.^33^ Briefly, the ascites samples underwent centrifugation at 1500 rpm for a duration of 5 min, after which the supernatant was removed. The resulting cell pellet was then incubated with 2 mL of red blood cell lysis buffer at ambient temperature for 5 min. For resected solid tissue, tumour specimens were isolated, washed and stored in ice‐cold DMEM containing 1% penicillin–streptomycin. The tumour was subsequently sectioned into pieces measuring 1–3 mm^3^ and subjected to centrifugation at a speed of 1500 rpm for a duration of 5 min. The resulting tissue was digested with an appropriate volume of tissue digestion solution in a 37°C shaking water bath for 30 min. The filtrate was collected using a 100 µM filter, and the supernatant was removed after centrifugation at 1500 rpm for 5 min. Isolated cells were resuspended in Matrigel (Corning) mixed with Ceturegel^®^ Matrix (Yeasen Biotechnology) at a ratio of 5:1 (Matrigel:medium). The droplets were initially placed on the dish and examined under a microscope. Following this, the dish was transferred to a 37°C incubator for a duration of 2 min. It was then inverted for 30 min to facilitate gel solidification. Afterwards, each well received an addition of 2 mL of organoid medium. The cultures were subsequently maintained at 37°C in an environment containing 5% CO_2_. OC organoids were successfully cultured and transfected with either control or LINC02776 knockdown lentivirus. They were subsequently plated in six‐well plates and maintained in culture for 5 days. Following this, they were exposed to either cisplatin or olaparib, or left untreated, for a duration of 48 h. Images of the organoids were captured using a CKX41 microscope (Olympus). To evaluate drug sensitivity, the viability of organoids was measured using the CellTiter‐Glo 3D cell viability assay (Promega), in accordance with the protocol provided by the manufacturer.

### 2.6 In vitro transcription and translation assays

Transcription and translation experiments were conducted following previously established protocols.^34^ To evaluate the coding capability of LINC02776 in vitro, we utilised the TnT Rapid Coupling Transcription/Translation System (Promega). Specifically, 2.0 µg of a circular plasmid harbouring the LINC02776 sequence under the control of a T7 promoter was introduced into the preprepared TnT Quick Master Mix. As a positive control, 2.0 µg of a T7‐promoter‐driven Luciferase plasmid was included. The reaction mixtures were incubated at ambient temperature for 90 min. Subsequently, the synthesised proteins were subjected to sodium dodecyl sulphate‐polyacrylamide gel electrophoresis (SDS‐PAGE) analysis and visualised using chemiluminescent detection.

### 2.7 Animal experiments

BALB/c mice were kept and cared for under specific pathogen‐free (SPF) conditions at the Laboratory Animal Center of FUSCC, adhering to institutional ethical guidelines throughout all animal experiments. To establish in vivo subcutaneous xenograft OC models, 1 × 10^6^ A2780 cells—either stably transfected with an empty vector or expressing LINC02776‐shRNA2—were dissociated using trypsin, resuspended in 200 µL of phosphate‐buffered saline (PBS), and injected into the right flanks of female BALB/c mice. Tumour growth and body weight were monitored every 3 days following implantation. At the end of 4 weeks, the animals were euthanised, and tumour volumes were determined using the formula: Volume = .5 × (Length × Width^2^). For in vivo drug efficacy studies, beginning 2 weeks postcell injection, mice received either cisplatin (5 mg/kg) or PBS via intraperitoneal administration every 3 days, totalling six injections. In experiments involving the in vivo delivery of LINC02776‐specific siRNA, once tumours reached approximately 100 mm^3^, mice were administered LINC02776‐specific siRNA or a nonspecific control siRNA (2 mg/kg per mouse) through tail vein injection. The siRNA transfection was facilitated by the in vivo‐jetPEI reagent (Polyplus‐transfection, Inc.), sourced from RIBOBIO.

### 2.8 Statistical analysis

Statistical evaluations were conducted utilising GraphPad Prism software (version 8.0). Results are expressed as means ± standard error of the mean (SEM), derived from a minimum of three independent experiments. For comparisons between two groups, an unpaired Student's *t*‐test was applied, whereas for comparisons among multiple groups, one‐way analysis of variance (ANOVA) was utilised. The relationship between overall survival (OS) and LINC02776 expression was assessed through Kaplan–Meier survival curves, with statistical significance evaluated via the log‐rank test. Cox proportional hazards modelling was employed to analyse risk factors impacting OC prognosis. Statistical significance was set at a *p*‐value threshold of less than .05.

## 3 RESULTS

### 3.1 LINC02776 is upregulated in platinum‐resistant OC patients

**FIGURE 1:**
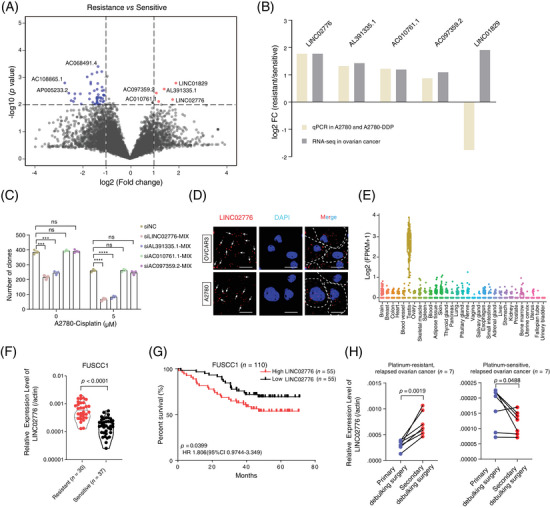
Contribution of LINC02776 to platinum resistance in ovarian cancer (OC) patients. (A) The volcano plot illustrates differentially expressed lncRNAs. The *X*‐axis represents log_2_‐transformed fold change values, while the *Y*‐axis represents ‐log_10_‐transformed *p*‐values. Red dots indicate significantly differentially expressed lncRNAs (*p* < .01 and |log_2_[fold change]| > 1). (B) Quantitative reverse transcription polymerase chain reaction (qRT‐PCR) validation of lncRNA candidate expression changes in A2780 and A2780‐DDP cells, compared to their RNA‐seq data. (C) Clonogenic assay showing colony formation efficiency in A2780 cells transfected with siNC, siLINC02776, siAL391335.1, siAC010761.1 or siAC097359.2, followed by treatment with 0 or 5 µM cisplatin for 14 days. (D) RNAscope assay showing for the subcellular localisation of LINC02776 (white dots, indicated by arrows) in OVCAR3 and A2780 cells. DAPI dihydrochloride (DAPI) (blue) stains the nucleus. Scale bar: 10 µm. (E) Expression levels of LINC02776 (in FPKM) across GTEx normal tissue RNA‐seq datasets. (F) Kaplan–Meier survival analysis showing the association between LINC02776 expression levels and overall survival of 110 OC patients. Patients were stratified into high‐ and low‐expression subgroups based on the median relative RNA abundance. (G) Comparison of LINC02776 expression levels between platinum‐resistant and platinum‐sensitive OC tissues. (H) qRT‐PCR analysis of LINC02776 expression in primary tumour tissues and paired secondary surgical tumour tissues from seven platinum‐resistant relapse patients and seven platinum‐sensitive relapse patients. Values are presented as mean ± standard error of the mean (SEM), *n* = 3 for panels (B–D and F–H). Statistical significance is indicated as follows: ns, not significant; ^*^ *p* < .05; ^**^ *p* < .01; ^***^ *p* < .001; ^****^ *p* < .0001.

To identify chemoresistance‐associated lncRNAs, we conducted a transcriptome‐wide analysis of RNA‐seq data derived from platinum‐sensitive (*n* = 10) and platinum‐resistant (*n* = 9) OC tissue samples (Table S2). A total of 8819 lncRNA genes (FPKM > .1 in at least two out of 19 samples) were analysed for differential expression using the EdgeR tool.^30^ This analysis revealed five upregulated and 46 downregulated lncRNAs in platinum‐resistant tissues (Figure 1A and Table S3). Functional enrichment analysis indicated that differentially expressed genes (DEGs) were primarily involved in pathways related to the regulation of drug metabolism, platinum drug resistance, pluripotency of stem cells, extracellular exosomes and cell proliferation (Figure S1A,B), supporting the reliability of our RNA‐seq data. To further validate these finding of differentially expressed lncRNAs, we established a cisplatin(DDP)‐resistant A2780 cell line (A2780‐DDP; Figure S1C). Expression levels of five upregulated lncRNAs (LINC02776, AL391335.1, AC010761.1, AC097359.2 and LINC01829) were compared between A2780‐DDP cells and their parental counterparts. With the exception of LINC01829, expression patterns of the remaining four lncRNAs aligned with the RNA‐seq data (Figure 1B). Among these, LINC02776 exhibited the highest fold‐change increase in both platinum‐resistant tissues and A2780‐DDP cells (Figure 1B). Moreover, LINC02776 knockdown significantly inhibited cell proliferation under cisplatin treatment (Figures 1C and S1D–F). Further characterisation of LINC02776 using RNAScope, RNA FISH and nucleocytoplasmic separation assays revealed that LINC02776 is distributed in both the cytoplasm and nucleus (Figures 1D and S1G,H). Tissue expression profiling indicated that LINC02776 showed low expression in normal tissues, except in the testis, highlighting its potential as a therapeutic target (Figures 1E and S1I). We then evaluated LINC02776 expression in OC tissues and cell lines, observing upregulation in platinum‐resistant patients and corresponding cisplatin‐resistant cell lines (A2780‐DDP and SKOV3‐DDP) (Figures 1F and S1J). Importantly, patients with higher LINC02776 levels exhibited shorter OS times (Figure 1G). Multivariate regression analyses confirmed that LINC02776 is an independent predictor of OS for OC patients (Figure S1K). Additionally, paired analysis of primary tumours and secondary surgical tumour tissues from seven platinum‐resistant relapse patients and seven platinum‐sensitive relapse patients demonstrated that LINC02776 was significantly upregulated in platinum‐resistant relapsed tumours (*p* = .0019) and downregulated in platinum‐sensitive relapsed tumours (*p* = .0488; Figure 1H). Collectively, these findings indicate that LINC02776 is significantly upregulated in platinum‐resistant OC and may serve as a promising prognostic biomarker and therapeutic target for OC patients.

### 3.2 Knockdown of LINC02776 inhibits OC cell proliferation

LINC02776 is located on chromosome 1q25.1 and expresses two primary isoforms, that is, isoform 1 (335 nucleotides, nt) and isoform 2 (659 nt; Figure S2A). These isoforms were further validated using 5′ and 3′ RACE assays (Figure S2B). Through quantitative reverse transcription PCR (qRT‐PCR) analysis, we found that LINC02776‐isoform 1 was highly expressed and significantly upregulated in cisplatin‐resistant cell lines (*p* < .0001, *p* = .002, respectively; Figure S2C). In contrast, LINC02776‐isoform 2 was significantly downregulated in cisplatin‐resistant cell lines (*p* = .0063, *p* = .0003, respectively; Figure S2D) but showed no significant differential expression in platinum‐resistant tissues (*p* = .4332; Figure S2E). To evaluate the protein‐coding potential of LINC02776, we used three computational tools: Coding Potential Assessment Tool^35^ (CPAT), CPC2 online tool^36^ (http://cpc.gao‐lab.org) and PhyloCSF codon substitution frequency analysis.^37^ All analysis indicated that LINC02776 lacks protein‐coding potential (Figure S3A,B). Additionally, in vitro transcription/translation assays confirmed that LINC02776 could be transcribed but not translated (Figure S3C). Collectively, these findings suggest that LINC02776‐isoform 1 (335 nt) is the predominantly expressed transcript in OC cells, significantly upregulated in cisplatin‐resistant cell lines, and exhibits no protein‐coding capability.

**FIGURE 2:**
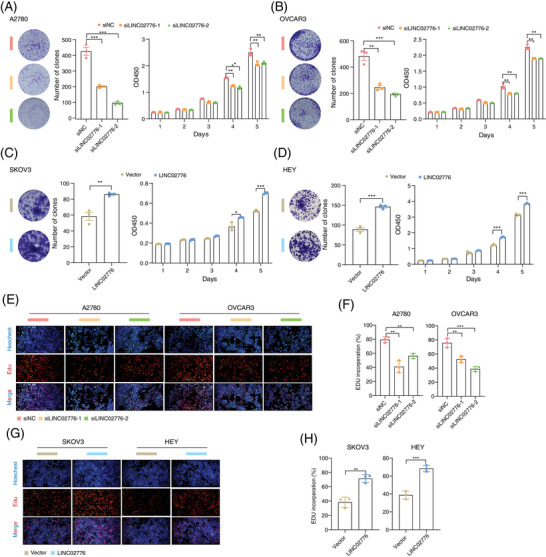
LINC02776 enhances ovarian cancer (OC) cell proliferation in vitro. (A, B) Colony formation assays (representative images) and CCK‐8 assays showing proliferation capacity in OVCAR3 cells and A2780 cells transfected with siRNA targeting LINC02776. (C, D) Colony formation assays (representative images) and CCK‐8 assays showing proliferation capacity in SKOV3 cells and HEY cells with stable LINC02776 overexpression induced by lentivirus transduction. (E, F) EdU incorporation assays showing representative images (E) and quantification (F) of DNA replication activity in A2780 and OVCAR3 cells transfected with siRNA against LINC02776. (G, H) EdU incorporation assays showing representative images (G) and quantification (H) of DNA replication activity in SKOV3 and HEY cells with stable overexpression of LINC02776 via lentivirus transduction. Images analysis was performed using Fiji software. Values are presented as mean ± standard error of the mean (SEM), *n* = 3 for all panels (A–H). Statistical significance is indicated as follow: ns, not significant; ^*^ *p* < .05; ^**^ *p* < .01; ^***^ *p* < .001; ^****^ *p* < .0001.

To further investigate the functional role of LINC02776 in OC cells, we silenced its expression using two siRNAs and ASOs in OC cell lines with high LINC02776 expression (Figure S1J). The knockdown efficiency of LINC02776 in A2780 and OVCAR3 cells was confirmed by qRT‐PCR (Figure S4A,B). Importantly, knockdown of LINC02776 had no effect on the expression of LINC02776‐isoform 2 or neighbouring genes *RC3H1* and *RC3H1‐IT1* (Figure S4C–E). Functional assays, including colony formation and Cell Counting Kit‐8 (CCK‐8) assays, revealed that LINC02776 knockdown significantly inhibited cell proliferation in A2780 and OVCAR3 cells (Figures 2A,B and S4F,G). Conversely, LINC02776 was overexpressed in SKOV3 and HEY cells, which have lower endogenous levels of LINC02776 (Figures S1J and S4H). Overexpression of LINC02776 significantly enhanced proliferation in both cell lines (Figure 2C,D). To explore the impact of LINC02776 on DNA replication, we performed EdU assays. Knockdown of LINC02776 significantly inhibited DNA replication (Figure 2E,F), while overexpression of LINC02776 promoted DNA replication (Figure 2G,H). To gain mechanistic insights, we conducted RNA‐seq enrichment analysis of LINC02776‐knockdown A2780 cells, which revealed significant enrichment of genes involved in DNA damage response (*p* < .0001) and apoptosis (*p* < .0001; Figure S4I,J and Table S6). These findings collectively indicate that LINC02776 promotes OC cell proliferation and DNA replication, and its knockdown impairs these processes. This suggests that LINC02776 may play a critical role in chemoresistance and represents a potential therapeutic target for OC.

### 3.3 LINC02776 modulates the sensitivity of OC cells to cisplatin by mitigating DNA damage

**FIGURE 3:**
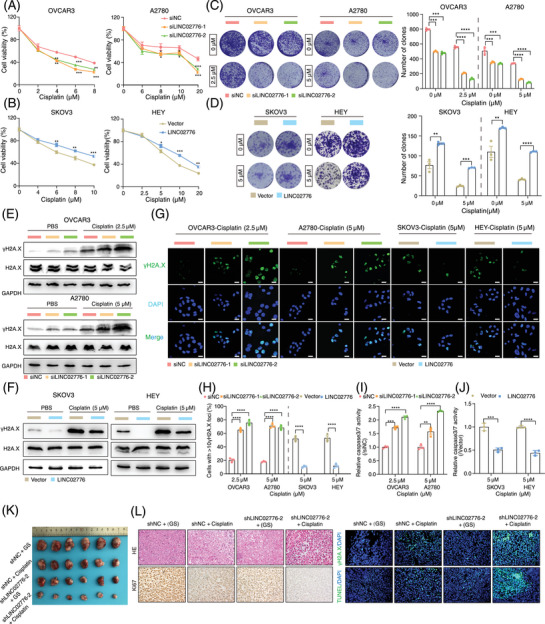
Knockdown of LINC02776 enhances cisplatin sensitivity in ovarian cancer (OC) cells in vitro and in vivo. (A) CCK‐8 assays showing cell viability in OVCAR3 (IC50 values are 6.12 µM, 3.68µM, 3.73 µM, respectively) and A2780 (IC50 values are 20.21µM, 11.56µM, 12.11µM, respectively) cells transfected with siNC or siLINC02776, treated with varying concentrations of cisplatin for 48 h. (B) CCK‐8 assays showing cell viability in SKOV3 (IC50 values are 7.82 µM, 10.13µM, respectively) and HEY (IC50 values are 8.53 µM, 12.53µM, respectively) cells transfected with Vector or LINC02776 overexpression construct, treated with varying concentrations of cisplatin for 48 h. (C) Colony formation assays with representative images and quantification in OVCAR3 and A2780 cells transfected with LINC02776‐siRNA, treated with varying concentrations of cisplatin for 2 weeks. Colonies were stained with crystal violet. (D) Colony formation assays with representative images and quantification in SKOV3 and HEY cells with stable LINC02776 overexpression, treated with varying concentrations of cisplatin for 3 weeks. Colonies were stained with crystal violet. (E, F) Western blot analysis showing γ‐H2A.X levels in OC cells treated with cisplatin for 24 h following LINC02776 knockdown (E) or overexpression (F). (G, H) Representative images (G) and quantification (H) of γ‐H2A.X foci staining in OVCAR3, A2780, SKOV3 and HEY cells treated with cisplatin for 24 h following LINC02776 knockdown or overexpression. Green indicates γ‐H2A.X; blue indicates DAPI. Scale bar: 20 µm. (I, J) Caspase 3/7 activity assays showing apoptotic activity in OC cells following LINC02776 knockdown (I) or overexpression (J). (K) Representative tumour images from A2780 xenograft models with LINC02776 knockdown, either alone or in combination with cisplatin. GS, 5% glucose solution. (L) Tumour sections stained with haematoxylin and eosin (H&E), anti‐Ki67 antibody, anti‐γ‐H2A.X antibody and TUNEL reagents to assess cell proliferation, DNA damage and apoptosis. Image analysis was performed using Fiji software. Values are presented as mean ± standard error of the mean (SEM), *n* = 3 for panels (A–J). Statistical significance is indicated as follows: ns, not significant; ^*^ *p* < .05; ^**^ *p* < .01; ^***^ *p* < .001; ^****^ *p* < .0001.

Cisplatin, a platinum‐based chemotherapeutic agent, exerts its antitumour effects by inducing DNA damage and inhibiting DNA synthesis, ultimately triggering tumour cell apoptosis. Our earlier results demonstrated that LINC02776 is significantly upregulated in platinum‐resistant OC tissues and cell lines. To further validate the role of LINC02776 in cisplatin resistance, we performed CCK‐8, colony formation and drug sensitivity assays. Knockdown of LINC02776 in OVCAR3 and A2780 cells enhanced their sensitivity to cisplatin (Figure 3A), whereas overexpression of LINC02776 in SKOV3 and HEY cells increased cisplatin resistance (Figure 3B). Under cisplatin treatment, LINC02776 knockdown significantly inhibited cell proliferation in OVCAR3 and A2780 cell lines (Figures 3C and S5A), while LINC02776 overexpression enhanced proliferation (Figures 3D and S5B). Furthermore, LINC02776 knockdown induced cell cycle arrest in the S phase (Figure S5C,D) and increased the percentage of Annexin V^+^ apoptotic cells (Figure S5E,F). Conversely, LINC02776 overexpression reduced apoptosis in SKOV3 and HEY cells (Figure S5G,H). These effects were also confirmed in cisplatin‐resistant OC cell lines (A2780‐DPP and SKOV3‐DDP) following LINC02776 silencing or overexpression (Figure S6A–C). Collectively, these findings indicate that LINC02776 knockdown promotes the cisplatin sensitivity in OC cells.

To determine whether LINC02776 contributes to cisplatin resistance by modulating DNA damage, we treated OVCAR3 and A2780 cells with cisplatin for 24 h after LINC02776 knockdown. Western blot analysis revealed a significant increase in γ‐H2A.X levels, a marker of DSB, in LINC02776‐knockdown cells (Figures 3E and S6D). In contrast, LINC02776 overexpression in SKOV3 and HEY cells reduced γ‐H2A.X levels (Figures 3F and S6E). Immunofluorescence staining further confirmed an increased number of γ‐H2A.X foci in LINC02776‐knockdown cells and a decreased number in LINC02776‐overexpressing cells (Figure 3G,H). Similar patterns were observed in cisplatin‐resistant OC cells (Figure S6F,G). In addition, we assessed caspase 3/7 activity, which is commonly elevated during apoptosis. Knockdown of LINC02776 increased caspase 3/7 activity in cisplatin‐treated cells (Figure 3I), whereas LINC02776 overexpression suppressed caspase 3/7 activity (Figure 3J). These results indicate that LINC02776 knockdown enhances DNA damage accumulation and promotes apoptosis, thereby increasing cisplatin sensitivity in OC cells.

To further evaluate whether LINC02776 targeting enhances cisplatin sensitivity in vivo, we established subcutaneous xenograft models using A2780 cells transfected with lentivirus‐mediated sh‐LINC02776. Mice were treated with either cisplatin or 5% glucose solution (GS) via intraperitoneal injection (Figure S7A). qRT‐PCR analysis confirmed a significant reduction in LINC02776 expression in the shLINC02776‐2 + GS and shLINC02776‐2 + cisplatin groups (*p* < .0001; Figure S7B). Tumour size and weight were significantly reduced in the LINC02776 knockdown group compared to controls (Figures 3K and S7C,D), suggesting that targeting LINC02776 suppresses tumour growth. Furthermore, cisplatin treatment in LINC02776‐deficient xenografts led to an increased γ‐H2A.X signal and enhanced apoptosis, while the Ki67 proliferation marker signal was reduced compared to controls (Figures 3L and S7E). These findings demonstrate that LINC02776 knockdown enhances cisplatin‐induced DNA damage and apoptosis, ultimately inhibiting tumour growth and improving cisplatin sensitivity. In summary, our study provides strong evidence that LINC02776 modulates cisplatin sensitivity in OC cells by regulating DNA damage and apoptosis pathways. Targeting LINC02776 represents a promising therapeutic strategy for overcoming platinum resistance in OC.

### 3.4 LINC02776 binds PARP1 to promote DNA damage‐triggered PARylation in OC cells

**FIGURE 4:**
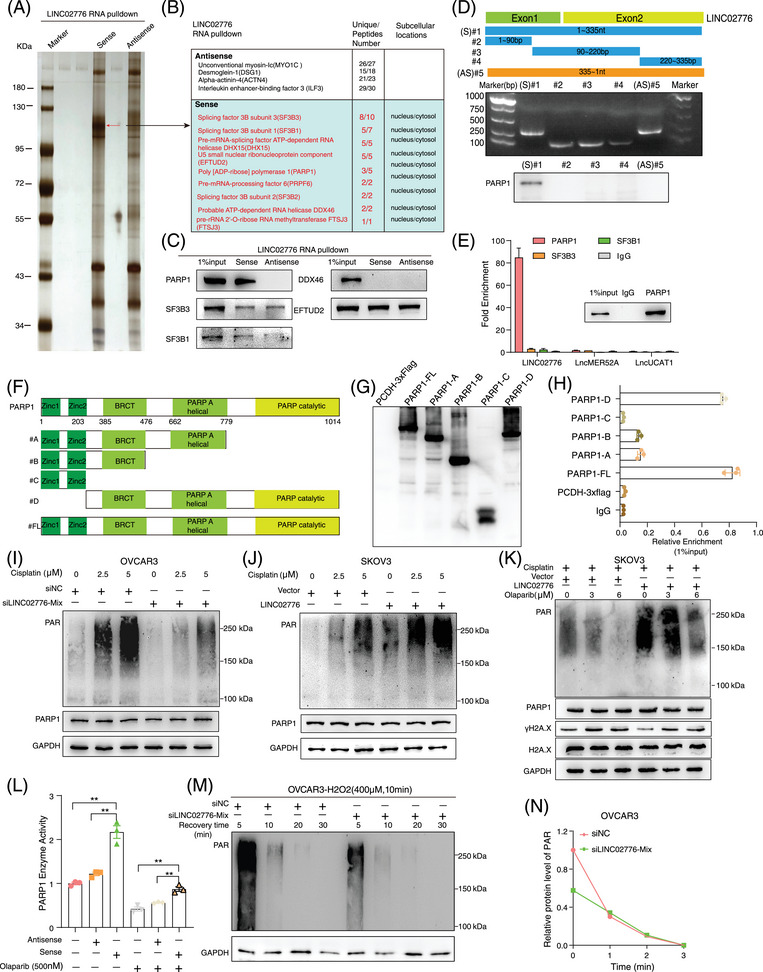
LINC02776 interacts with PARP1 to promote DNA damage‐triggered polyADP‐ribosylation (PARylation) in ovarian cancer (OC) cells. (A) RNA pull‐down assay showing proteins bound to biotinylated LINC02776 in vitro, visualised by silver staining. (B) Top proteins identified through mass spectrometric (MS) analysis following RNA pull‐down. (C) Western blot validation of RNA pull‐down assays using biotin‐labelled sense and antisense probes for LINC02776. (D) Western blot analysis detecting PARP1 proteins pulled down by in vitro‐transcribed biotinylated RNAs, corresponding to full‐length LINC02776 and its three truncated fragments. (E) RIP assay using an anti‐PARP1 antibody, followed by quantitative reverse transcription polymerase chain reaction (qRT‐PCR) to quantify LINC02776 enrichment. LncMER52A and LncUCAT1 served as negative controls. (F) Schematic illustration of wild‐type PARP1 and its deletion mutants, highlighting key structural domains: two zinc‐finger motifs (Zinc1, Zinc2), an automodification domain (BRCA1 C‐Terminus (BRCT) domain) (BRCA1 C‐Terminus), a helical domain and the PARP catalytic motif. (G) Western blot validation of wild‐type PARP1 and its truncated mutants. (H) RIP assay detecting LINC02776 enrichment in full‐length PARP1 and its truncated mutants in vitro. (I, J) Western blot analysis of PAR levels in OVCAR3 cells transfected with LINC02776‐siRNA (I) and in LINC02776‐overexpressing SKOV3 cells (J) treated with 2.5 µM or 5 µM cisplatin for 24 h. (K) Western blot analysis of PAR, PARP1, γ‐H2A.X, H2A.X and GAPDH levels in LINC02776‐overexpressing SKOV3 cells treated with various concentrations of olaparib for 4 h, followed by 5 µM cisplatin treatment for 24 h. (L) Enzymatic activity assay showing the activity of human recombinant PARP1 in the presence or absence of LINC02776. (M) Western blot analysis of PAR levels in OVCAR3 cells transfected with LINC02776‐siRNA, treated with 400 µM H_2_O_2_ for 10 min. After replacing the medium, cell lysates were collected at specific time points (5, 10, 20 and 30 min) to measure PAR levels. (N) Quantification of PAR protein levels from panel. (M) Image analysis was conducted using Fiji software. Values are presented as mean ± standard error of the mean (SEM), *n* = 3 for panels (C, E, H and I). Statistical significance is indicated as follows: ns, not significant; ^*^ *p* < .05; ^**^ *p* < .01; ^***^ *p* < .001; ^****^ *p* < .0001.

To elucidate the mechanism by which LINC02776 contributes to cisplatin resistance in OC cells, we conducted biotin‐labelled RNA pull‐down assays followed by mass spectrometric (MS) analysis (Figure 4A). Specific protein bands associated with LINC02776 RNA were identified by MS, and PARP1 was found to interact specifically with LINC02776 (Figure 4B,C). This interaction was further validated in A2780‐DDP cells (Figure S8A), with antisense strands of the same length but different secondary structures serving as negative controls. To identify the essential binding region of LINC02776 for PARP1 interaction, we generated a series of truncated LINC02776 fragments based on its predicted secondary structure using RNAfold (Figures 4D and S8B). The RNA pull‐down assay demonstrated that the full‐length LINC02776 sequence was essential for binding to PARP1 (Figure 4D). To confirm the interaction, we performed RIP assays using an anti‐PARP1 antibody, which revealed a significant enrichment of LINC02776 with PARP1 (Figure 4E). Further RIP assays pinpointed the 779–1014 amino acid (aa) region of PARP1 as the primary interaction site for LINC02776 binding (Figure 4F–H). Immunofluorescence assays revealed colocalisation of LINC02776 and PARP1 in OC cells (Figure S8C). These findings collectively indicate that LINC02776 directly interacts with PARP1 in OC cells.

To investigate the functional consequences of the LINC02776–PARP1 interaction, we assessed whether LINC02776 knockdown or overexpression affects PARP1 expression. Neither LINC02776 knockdown nor overexpression altered PARP1 mRNA or protein levels (Figure S9A,B). Similarly, manipulating PARP1 expression did not influence LINC02776 levels (Figure S9C,D). PARP1 is known to mediate DNA repair through PARylation, a process in which PAR polymers are synthesised using NAD^+^ and transferred to acceptor proteins, including PARP1 itself.^17^, ^38^ Since LINC02776 binds to the catalytic domain of PARP1, we examined whether PARylation affects this interaction. RNA pull‐down and RIP assays revealed that treatment with olaparib, a PARP1 inhibitor, significantly disrupt the LINC02776–PARP1 interaction in both OVCAR3 and A2780 cells (Figure S10A,B). These findings suggest that the interaction between LINC02776 and PARP1 is dependent on PARylatin activity. Next, we hypothesised that LINC02776 modulates PARP1‐mediated PARylation activity. Following cisplatin treatment, LINC02776 knockdown significantly reduced PARylated protein levels in OVCAR3 and A2780 cells (Figures 4I and S10C,D), while LINC02776 overexpression markedly increased PARylation levels in SKOV3 and HEY cells (Figures 4J and S10E,F). Furthermore, higher concentrations of olaparib were required to inhibit PARylation in LINC02776‐overexpressing cells (Figures 4K and S10G,H). PARP1 enzymatic activity assays confirmed that LINC02776 overexpression enhanced PARP1 activity (Figures 4L and S10I).

To further examine the dynamics of PARylation activity, we exposed OVCAR3 and A2780 cells to H_2_O_2_, a well‐known DNA damage inducer. PARylation proteins increased significantly after H_2_O_2_ exposure; however, the damage‐induced PAR signal was markedly reduced in LINC02776‐depleted cells (Figure S10J). Notably, although control cells (siNC) showed rapid PARylation induction following H_2_O_2_ treatment, PARylation levels returned to baseline within 30 min in both control and knockdown groups (Figures 4 M,N and S10K,L). This suggests that LINC02776 does not affect dePARylation activity, but rather enhances PARylation initiation and maintenance. Given the previously reported association between elevated PARylation levels and cisplatin resistance,^39^, ^40^, ^41^ we further validated these findings in OC tissues via western blot analysis (Figure S10M). Importantly, in SKOV3 and HEY cells overexpressing LINC02776, combined treatment with cisplatin and olaparib abolished the difference in cisplatin sensitivity between control and overexpressing cells (Figure S10N). Collectively, these findings reveal a novel function of LINC02776 in facilitating PARP1‐mediated PARylation, thereby reducing DNA damage and promoting cisplatin resistance in OC cells. Targeting the LINC02776–PARP1 interaction may represent a promising therapeutic strategy for overcoming platinum resistance in OC.

### 3.5 LINC02776 promotes drug resistance through homologous recombination restoration

**FIGURE 5:**
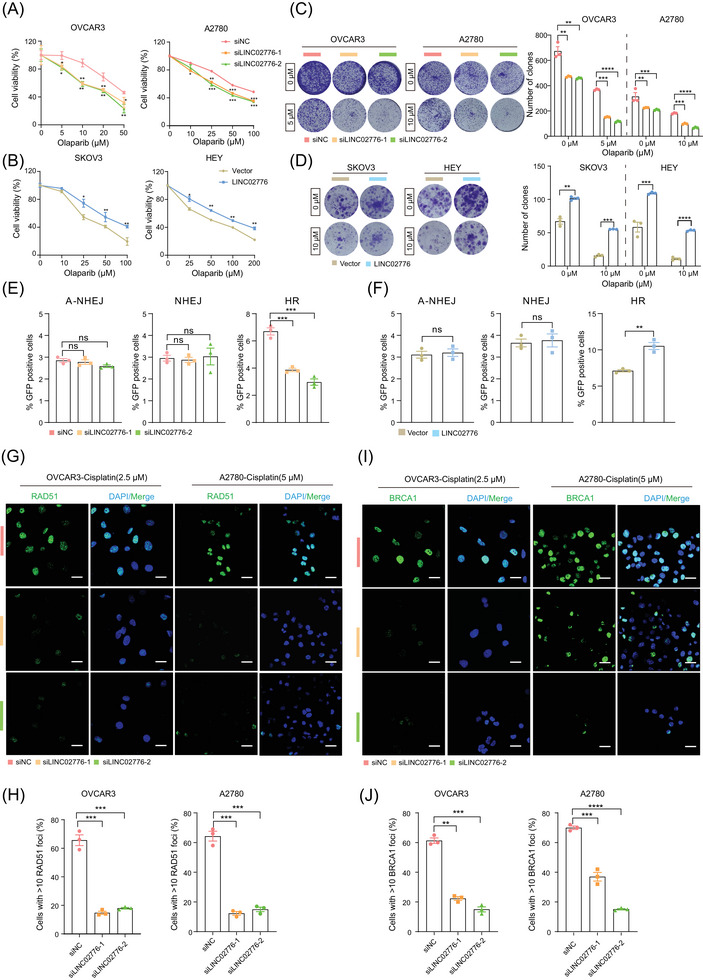
LINC02776 promotes olaparib resistance and homologous recombination (HR) restoration. (A) CCK‐8 assays showing cell viability in OVCAR3 (IC_50_ values are 24.63 µM, 16.17 µM, 15.51 µM, respectively) and A2780 (IC_50_ values are 60.15 µM, 36.81 µM, 35.04 µM, respectively) cells transfected with siNC or siLINC02776, treated with varying concentrations of olaparib for 48 h. (B) CCK‐8 assays showing cell viability in SKOV3 (IC_50_ values are 30.98 µM, 55.76 µM, respectively) and HEY (IC_50_ values are 57.25 µM, 102.0 µM, respectively) cells, transfected with Vector or LINC02776 overexpression construct, treated with varying concentrations of olaparib for 48 h. (C, D) Colony formation assays with representative images and quantification in A2780 and OVCAR3 cells transfected with LINC02776‐siRNA (C) and SKOV3 and HEY cells with stable LINC02776 overexpression (D), treated with varying concentrations of olaparib for 2–3 weeks. Colonies were stained with crystal violet. (E, F) Assessment of A‐nonhomologous end joining (NHEJ), NHEJ and HR repair pathway efficiency in U2OS cells carrying EJ2‐GFP, EJ5‐GFP and DR‐GFP reporter constructs transfected with LINC02776‐siRNAs (E) or LINC02776 overexpression vector (F). (G, H) Representative images (G) and quantification (H) of RAD51 nuclear foci formation in OVCAR3 and A2780 cells transfected with LINC02776‐siRNA, following 24 h of cisplatin treatment. Green indicates RAD51; blue indicates DAPI. Scale bar: 20 µm. (I, J) Representative images (I) and quantification (J) of BRCA1 nuclear foci formation in OVCAR3 and A2780 cells transfected with LINC02776‐siRNA, following 24 h of cisplatin treatment. Green indicates BRCA1; blue indicates DAPI. Scale bar: 20 µm. Quantification: The percentages of cells with more than 10 nuclear foci are presented as mean ± standard error of the mean (SEM). Values are presented as mean ± SEM, *n* = 3 for all panels (A–J). Statistical significance is indicated as follows: ns, not significant; ^*^ *p* < .05; ^**^ *p* < .01; ^***^ *p* < .001; ^****^ *p* < .0001.

The expression of LINC02776 was shown to enhance DNA damage‐induced PARP1 activation, indicating that higher concentrations of olaparib might be required to effectively inhibit PARP1 activity in LINC02776‐overexpressing OC cells (Figure 4). Additionally, LINC02776 knockdown significantly increased the levels of chromatin‐bound PARP1, suggesting that LINC02776 prevents olaparib‐induced PARP1 trapping at DNA damage sites (Figure S10O). To determine whether LINC02776 expression affects olaparib resistance in OC cells, we performed CCK‐8, colony formation and drug sensitivity assays. Knockdown of LINC02776 significantly increased the sensitivity of OC cells to olaparib (Figure 5A,C), while overexpression of LINC02776 resulted in reduced sensitivity to olaparib (Figure 5B,D). PARP1 is a key player in DSB repair, participating in pathways such as A‐NHEJ, NHEJ and HR.^17^ To identify which pathway is regulated by LINC02776, we employed U2OS cell lines containing GFP reporter vectors specific for HR, NHEJ and A‐NHEJ repair pathways. Silencing LINC02776 significantly decreased the frequency of HR repair, with no apparent effect on NHEJ or A‐NHEJ repair efficiency (Figure 5E). Conversely, LINC02776 overexpression significantly increased of HR repair efficiency (Figure 5F). Given PARP1's established role in HR pathway regulation, including its involvement in recruiting key HR‐related proteins such as BRCA1 and RAD51,^42^, ^43^, ^44^ we further examine the impact of LINC02776 on the recruitment of these proteins. The repression of LINC02776 reduced the recruitment of BRCA1 and RAD51 to chromatin after cisplatin treatment in OC cells (Figure 5G–J). These findings suggest that LINC02776 mediates cisplatin and olaparib resistance in OC cells by enhancing PARP1‐mediated PARylation activity and promoting HR through the recruitment of BRCA1 and RAD51. Collectively, these results highlight LINC02776 as a key modulator of HR repair efficiency and a potential therapeutic target for overcoming platinum and PARP inhibitor resistance in OC.

### 3.6 HIF‐1α is essential for modulating LINC02776 transcription

**FIGURE 6:**
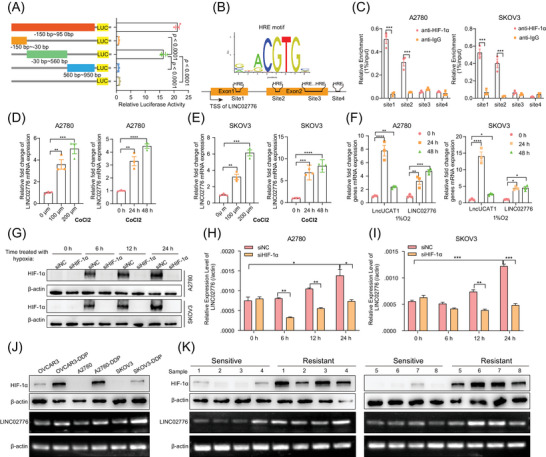
LINC02776 is transcriptionally induced by HIF‐1α under hypoxia. (A) Identification of the core promoter region of LINC02776. The left panel shows a schematic diagram of the 1.1 kb full‐length promoter and its truncated fragments. The right panel displays luciferase activity of the full‐length and truncated promoter fragments in HEK293T cells. (B) Schematic representation of hypoxia response elements (HREs) located within the core promoter region of LINC02776. (C) ChIP assay demonstrating the binding capacity of HIF‐1α to each HRE site in A2780 and SKOV3 cells after 24 h of hypoxia treatment. (D, E) Relative expression of LINC02776 measured by quantitative reverse transcription polymerase chain reaction (qRT‐PCR) in A2780 (D) and SKOV3 (E) cells treated with different concentrations of CoCl_2_, a chemical hypoxia inducer. (F) Relative expression of LINC02776 measured by qRT‐PCR in A2780 and SKOV3 cells cultured under 1% O_2_ hypoxic conditions. LncUCAT1 was used as a positive control. (G) Western blot analysis showing HIF‐1α protein levels in A2780 and SKOV3 cells transfected with siNC or HIF‐1α siRNAs under hypoxic conditions at different time points. (H, I) qRT‐PCR analysis showing relative expression of LINC02776 in A2780 (H) and SKOV3 (I) cells transfected with siNC or HIF‐1α siRNAs under hypoxic conditions at different time points. (J, K) Comparison of LINC02776 and HIF‐1α expression levels between platinum‐resistant and platinum‐sensitive ovarian cancer (OC) cell lines (J) and tissues (K). Values are presented as mean ± standard error of the mean (SEM), *n* = 3 for panels (A, C–I). Statistical significance is indicated as follows: ns, not significant; ^*^ *p* < .05; ^**^ *p* < .01; ^***^ *p* < .001; ^****^ *p* < .0001.

To investigate the mechanism underlying the upregulation of LINC02776 in platinum‐resistant OC cells and tissues, we analysed the LINC02776 promoter sequence (−150 to +950 bp from the transcription start site) using data from the FANTOM5 database.^45^ Both the full‐length and truncated promoter DNA fragments were cloned into a luciferase reporter vector. Luciferase assays revealed that the −30 to +560 bp region exhibited significantly higher luciferase activity than other truncated fragments (*p* < .0001, Figure 6A). This region was identified as the core promoter region of LINC02776, and it contained multiple hypoxia response elements (HREs; Figure 6B). To validate the role of HREs in LINC02776 transcriptional regulation, we performed ChIP assays, which demonstrated that HIF‐1α primarily binds to the first and second HRE sites within the promoter region (Figure 6C). To further confirm whether hypoxia regulates LINC02776 transcription, OC cells were treated with CoCl_2_, a chemical inducer of hypoxia‐inducible factor (HIF). LINC02776 expression was upregulated in a dose‐ and time‐dependent manner following CoCl_2_ treatment (Figure 6D,E). As a positive control, LncUCAT1, a known hypoxia‐induced lncRNA,^46^ also showed increased expression. In addition, OC cells were cultured under 1% O_2_ hypoxic conditions, resulting in a time‐dependent increase in LINC02776 expression (Figure 6F). However, when HIF‐1α was knocked down under hypoxic conditions, the upregulation of LINC02776 was significantly reduced (Figure 6G–I). In platinum‐resistant OC cells and tissues, we observed higher HIF‐1α protein expression levels compared to platinum‐sensitive controls (Figure 6J,K), consistent with previous reports.^47^, ^48^, ^49^ These findings collectively demonstrate that HIF‐1α directly regulates LINC02776 transcription by binding to HRE sites in its promoter region, contributing to the upregulation of LINC02776 in platinum‐resistant OC cells and tissues.

### 3.7 Targeting LINC02776 inhibits OC cell growth and reverses drug resistance in vivo

**FIGURE 7:**
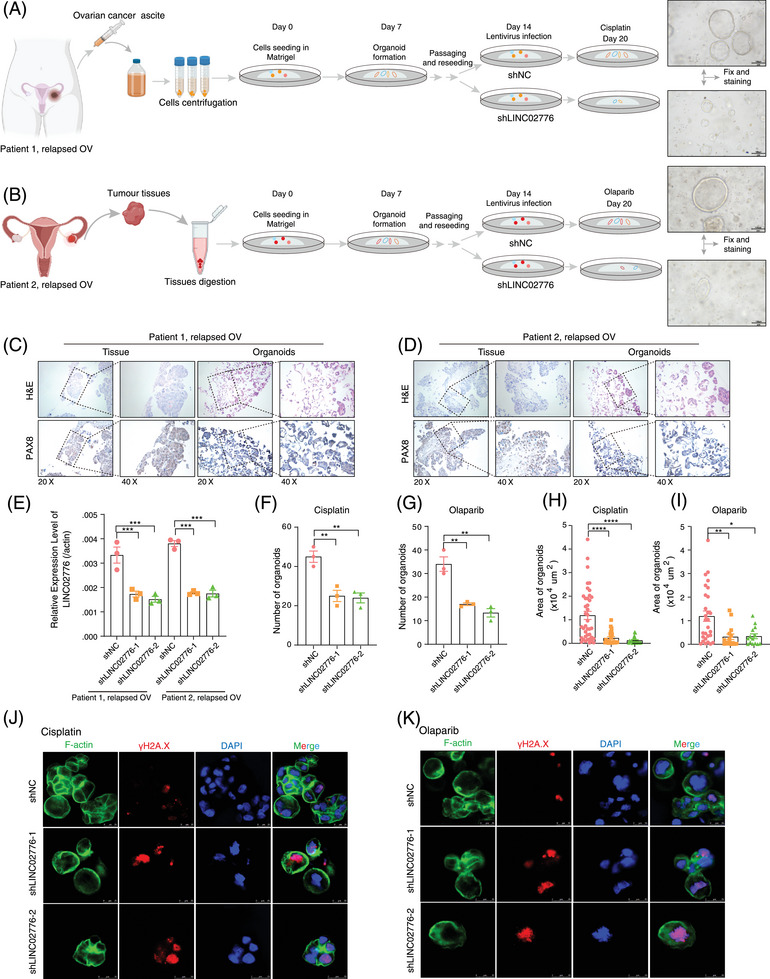
LINC02776 knockdown inhibits ovarian cancer (OC) cell growth and reverses drug resistance in OC patient‐derived organoid (PDO) models. (A, B) Schematic illustration showing the establishment and subsequent treatments of platinum‐resistant (A) and olaparib‐resistant (B) patient‐derived OC organoids. (C, D) Histological comparison between OC organoids and their corresponding tumour tissue. The top panels show haematoxylin and eosin (H&E) staining, while the bottom panels display PAX8 staining, highlighting the similarity in histological features. (E) Quantitative reverse transcription polymerase chain reaction (qRT‐PCR) analysis showing the mRNA expression levels of LINC02776 in OC organoids transfected with two independent shRNAs targeting LINC02776. (F, G) LINC02776 knockdown reduced the number of OC organoids following treatment with cisplatin (F) or olaparib (G), indicating increased drug sensitivity. (H, I) LINC02776 knockdown decreased the size of OC organoids after treatment with cisplatin (H) or olaparib (I), further supporting its role in drug resistance. (J, K) Representative images of control organoids and LINC02776 shRNAs‐transfected organoids, stained with F‐actin (green), γ‐H2A.X (red) and DAPI (blue), highlighting increased DNA damage (γ‐H2A.X foci) in LINC02776‐depleted organoids. Values are presented as mean ± standard error of the mean (SEM). Statistical significance is indicated as follows: ns, not significant; ^*^ *p* < .05; ^**^ *p* < .01; ^***^ *p* < .001; ^****^ *p* < .0001.

To further validate the critical role of LINC02776 in drug resistance, tumour tissues and ascites samples were collected from OC patients who underwent tumour resection or ascites drainage. PDOs derived from patient tissues, exhibiting diverse drug resistance characteristics, were successfully developed from the collected samples. Specifically, the tumour tissues were suspended in a basement membrane extract, then plated and cultured using a medium specifically formulated for organoid growth (as shown in Figure 7A,B). Histological evaluation through haematoxylin and eosin (H&E) staining and immunohistochemistry (IHC) verified the expression of critical OC protein markers, including PAX8.^50^ The histopathological features of PDOs closely resembled their parental tumour tissues (Figure 7C,D). To investigate the functional role of LINC02776 in drug resistance, OC organoids were transduced with lentiviruses carrying shRNAs targeting LINC02776, leading to efficient knockdown (Figure 7E). Following treatment with cisplatin or olaparib, organoids in the LINC02776 knockdown group exhibited fewer and smaller colonies to the control group (Figure 7F–I). Viability assays using the CellTiter‐Glo 3D Assay revealed that LINC02776 knockdown significantly enhanced the cytotoxic effects of cisplatin and olaparib (Figure S11A,B). Additionally, γ‐H2A.X staining, a marker of DNA DSBs, showed a dramatic increase in γ‐H2A.X foci in the LINC02776 knockdown group, indicating impaired DSB repair (Figure 7J,K). These results suggest that LINC02776 knockdown enhances cisplatin and olaparib sensitivity in OC PDO models by preventing efficient DNA damage repair.

**FIGURE 8:**
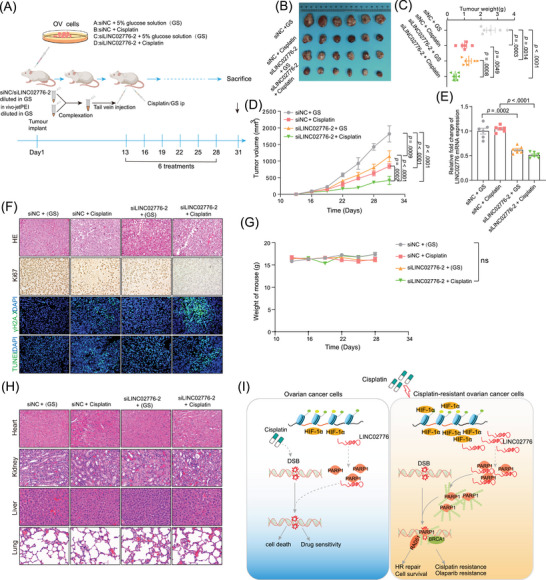
Targeting LINC02776 exhibits antitumour potential. (A) Schematic illustration of the in vivo treatment procedure, showing the administration of LINC02776 siRNA using in vivo‐jetPEI, either alone or in combination with cisplatin, in a subcutaneous xenograft mouse model. (B) Representative images of A2780 xenograft tumours harvested from mice following the indicated treatments. (C, D) Quantification of tumour weights (C) and tumour volumes (D) from mice treated as indicated in (A). *n* = 6 per group, analysed using a two‐sided Student's *t*‐test. (E) Quantitative reverse transcription polymerase chain reaction (qRT‐PCR) analysis showing LINC02776 mRNA expression levels in xenograft tumour tissues from each treatment group. (F) Histological and immunohistochemical analysis of tumour sections from panel (B), stained with haematoxylin and eosin (H&E), anti‐Ki67 antibody, anti‐γ‐H2A.X antibody and TUNEL reagents, assessing cell proliferation, DNA damage and apoptosis. (G) Body weight monitoring of mice throughout the experimental period, measured every other day (*n* = 6 per group), showing no significant differences between treatment groups. (H) Representative H&E staining images of major organs (heart, liver, lungs and kidneys), collected from mice at the end of the experiment, showing no significant histopathological toxicity in any group. Image analysis was performed using Fiji software. Values are presented as mean ± standard error of the mean (SEM). Statistical significance is indicated as follows: ns, not significant; ^*^ *p* < .05; ^**^ *p* < .01; ^***^ *p* < .001; ^****^ *p* < .0001.

To further explore the potential of LINC02776 as an antitumour target, we used in vivo‐jetPEI to deliver LINC02776‐specific siRNA via tail vein injection into mice bearing A2780 xenografts (Figure 8A). Among all treatment groups, the siLINC02776‐2 + cisplatin group exhibited the greatest antitumour effect, with significant reductions in tumour volume and weight (Figure 8B–D). qRT‐PCR analysis confirmed a significant reduction in LINC02776 expression in the siLINC02776‐2 + GS and siLINC02776‐2 + cisplatin groups (*p* = .0002, *p* < .0001, respectively, Figure 8E). Furthermore, IHC staining and IF revealed a significant increase in apoptotic cells (TUNEL assay) and a decrease in Ki67‐positive proliferating cells in the siLINC02776‐2 + cisplatin group compared to controls (Figures 8F and S11C). These results indicate a synergistic antitumour effect between LINC02776 knockdown and cisplatin treatment. To assess the systemic toxicity of the combined therapy, we monitored body weight changes in the mice and observed no significant differences between groups, suggesting an absence of systemic toxicity at the administered doses (Figure 8G). Additionally, H&E staining of major organs (heart, liver, lung and kidney) revealed no evidence of histopathological toxicity (Figure 8H). In summary, our findings demonstrate that LINC02776 knockdown enhances cisplatin and olaparib sensitivity in PDO and xenograft models by disrupting DNA damage repair mechanisms. Mechanistically, LINC02776 promotes PARP1‐mediated PARylation, facilitating DNA repair efficiency and contributing to platinum resistance in OC cells (Figure 8I). Importantly, LINC02776 emerges as a promising therapeutic target for overcoming chemotherapy resistance in OC patients.

## 4 DISCUSSION

A substantial body of research has significantly advanced our understanding of the molecular mechanisms underlying chemotherapy resistance in cancer patients. However, the specific regulatory pathways remain poorly defined, particularly in OC. In this study, we screened for potential lncRNAs associated with platinum resistance in OC and identified LINC02776 as significantly upregulated in platinum‐resistant OC patients compared to those sensitive to platinum‐based therapies. Mechanistically, we demonstrated that LINC02776 directly interacts with PARP1, enhancing PARP1‐mediated PARylation. This interaction increases HR repair efficiency and promotes the recruitment of key DNA repair factors, including BRCA1 and RAD51, to sites of DNA damage. These molecular events collectively contribute to cisplatin resistance in OC cells.

In recent years, lncRNAs have emerged as critical regulators of various biological processes, including tumour proliferation, metastasis, metabolism and notably, drug resistance.^20^, ^21^ Due to their tissue‐specific and disease‐specific expression patterns, lncRNAs are gaining recognition as promising biomarkers and therapeutic targets, with the potential to selectively affect diseased cells.^51^, ^52^ In our previous research, we identified LncMER52A as a liver cancer‐specific lncRNA, where its targeting successfully inhibited tumour metastasis.^34^ In the current study, we observed that LINC02776 is selectively overexpressed in platinum‐resistant OC tissues, with minimal expression in normal tissues except for the testis. This overexpression correlates with poor prognosis in OC patients. Furthermore, LINC02776 expression levels were elevated in platinum‐resistant relapsed patients compared to platinum‐sensitive relapsed patients. These findings suggest that LINC02776 represents a viable therapeutic target for platinum‐resistant OC, given its tumour‐selective expression profile. In both OC cell lines and PDO models, we demonstrated that silencing LINC02776 significantly inhibited cell proliferation and promoted apoptosis. Importantly, LINC02776 knockdown enhanced cisplatin sensitivity and increased γ‐H2A.X accumulation, indicating elevated DNA damage.

To elucidate the mechanism by which LINC02776 promotes platinum resistance, we discovered that LINC02776 directly binds to the catalytic domain of PARP1. Further experiments revealed that neither LINC02776 nor PARP1 altered each other's mRNA or protein expression levels. However, LINC02776 overexpression significantly elevated total cellular PARylation levels in cisplatin‐treated OC cells, whereas LINC02776 knockdown markedly reduced these levels. Since PARylated proteins are the products of PARP1‐catalysed auto‐PARylation and transmodification of substrate proteins,^53^ we hypothesised that LINC02776 might influence the enzymatic activity of PARP1. To test this, we performed an in vitro PARP1 enzyme activity assay, confirming that LINC02776 enhances PARP1 enzymatic activity without affecting the activity of de‐PARylating enzymes. Notably, the LINC02776‐mediated increase in PAR levels was transient, as it was no longer observed 30 min after H_2_O_2_ treatment. This finding suggests that LINC02776 does not alter the kinetics of de‐PARylation, but instead primarily impacts PARP1 activation and PARylation initiation. PARylation is a dynamic post‐translational modification that regulates critical cellular processes, including DNA damage detection and repair, cell death and transcriptional activity.^54^, ^55^ Activated PARP1 serves as a scaffold to recruit essential DNA repair factors to sites of damage, facilitating DNA damage repair.^53^ Furthermore, PARP1 modulates multiple DNA repair pathways,^56^ including SSB repair, BER, NER and DSB repair via HR, NHEJ and A‐NHEJ pathways. In our study, we observed that LINC02776 promotes OC cell proliferation primarily by modulating the HR pathway. Immunofluorescence staining revealed that silencing LINC02776 reduced the recruitment and average fluorescence intensity of key HR‐related proteins, including BRCA1 and RAD51, at sites of DNA damage. These findings suggest that LINC02776 enhances HR‐mediated DNA repair efficiency, contributing to platinum resistance and sustained OC cell proliferation under chemotherapeutic stress.

Under cisplatin treatment, our observations indicated that increased levels of LINC02776 necessitated greater concentrations of the PARP inhibitor olaparib to efficiently inhibit DNA damage‐induced PARP1 activity. This finding suggests that LINC02776 modulates the sensitivity of OC cells to PARP inhibitors. Previous studies have shown that PARPi resistance can arise from increased PARP1 enzymatic activity. For example, HMGA2, which belongs to the HMG protein family, has been shown to increase the PARylation activity of PARP1 by directly interacting with it, thereby contributing to resistance against PARPi.^57^ Similarly, HMGB3 directly interacts with PARP1 to enhance PARylation activity, promoting PARPi resistance in OC cells.^58^ Additionally, Lnc15.2/PACMP regulates PARP1‐dependent PARylation in response to DNA damage, further contributing to PARPi resistance.^59^ In our study, LINC02776 depletion in OC cells and PDO models reversed PARPi resistance, highlighting its role in PARPi sensitivity modulation. Notably, targeting LINC02776 increased the sensitivity of OC cells to both cisplatin and olaparib in both in vivo and in vitro OC models. In recent years, PDO technology has become a critical tool in precision oncology research. This technology allows for the long‐term cultivation of cancer cells derived from patients in vitro while accurately maintaining their in vivo phenotypes.^60^, ^61^ PDOs have been successfully developed across various types of cancer, including prostate,^62^ pancreas,^63^ breast,^64^ liver,^65^ gastrointestinal tract^66^ and ovary.^33^ These models facilitate a deeper understanding of the phenotypic and molecular heterogeneity of tumours. Importantly, PDO chemoresistance correlates with clinical treatment outcomes,^67^, ^68^ positioning PDOs as reliable surrogates for assessing chemotherapy sensitivity. In our study, we established OC‐derived PDOs exhibiting resistance to platinum and olaparib. ShRNA‐mediated depletion of LINC02776 in these PDOs significantly enhanced chemosensitivity to both drugs. Moreover, in vivo inhibition of LINC02776 using siRNA demonstrated similar effects, further validating its role as a therapeutic target for overcoming drug resistance. In conclusion, our findings reveal that LINC02776 is overexpressed in platinum‐resistant tissues and that its elevated expression correlates with poor prognosis. Mechanistically, LINC02776 promotes platinum and PARPi resistance by directly interacting with PARP1 and enhancing its enzymatic activity, facilitating DNA damage repair via HR. Importantly, targeting LINC02776 offers a promising therapeutic strategy to overcome chemoresistance in OC patients, potentially improving their response to both platinum‐based therapies and PARP inhibitors.

## AUTHOR CONTRIBUTIONS

Conception and design: Yangjun Wu, Xiaohua Wu, Shengli Li, Hao Wen. Development of methodology: Yangjun Wu, Yu Zeng, Yong Wu Acquisition of data (provided organoid, animals, acquired and managed patients, provided facilities, etc.): Yangjun Wu, Bin Zheng, Lulu Yang, Jun Wang, Zheng Feng, Xingzhu Ju, Hao Wen. Analysis and interpretation of data (e.g., statistical analysis, biostatistics, computational analysis): Yangjun Wu, Yu Zeng, Yong Wu, Shengli Li, Hao Wen. Writing, review, and/or revision of the manuscript: Yangjun Wu, Xinyu Ha, Shengli Li. Administrative, technical, or material support (i.e., reporting or organizing data, constructing databases): Yangjun Wu,Xinyu Ha, Chaohua Liu, Ziqi Liu, Y. Jiajia Wang. Shenglin Huang, Linhui Liang, Xiaohua Wu, Shengli Li, Hao Wen. Study supervision: Yangjun Wu, Xiaohua Wu, Shengli Li, Hao Wen.

## CONFLICT OF INTEREST STATEMENT

The authors declare no conflicts of interest.

## ETHICS STATEMENT

Fresh ovarian cancer tissue samples were collected from surgical specimens at the Department of Gynecologic Oncology, FUSCC, after obtaining written informed consent from all participants. The study received ethical approval from the Medical Ethical Committee of FUSCC (Approval No. 2110244‐9). All animal studies adhered to the guidelines set by Fudan University for animal research and were approved by the Animal Care Committee of Fudan University.

## Supporting information



Supporting Information

Supporting Information

Supporting Information

## Data Availability

The raw RNA‐seq data obtained in this study have been uploaded to the GEO database, accessible under the accession number GSE214302. Further data that support the findings of this research can be obtained from the corresponding authors upon reasonable request.
