# Supplementary material for: HIF‐1α‐induced long noncoding RNA LINC02776 promotes drug resistance of ovarian cancer by increasing polyADP‐ribosylation
Source: Clin Transl Med. 2025 Mar 21;15(3):e70244. doi: 10.1002/ctm2.70244 (PMC11928293; doi:10.1002/ctm2.70244)
Supplement: Supplementary file 1 — Supporting Information [file CTM2-15-e70244-s002.pdf]

Supplementary Materials and Methods

Cell culture

Ovarian cancer cell lines were obtained from the American Type Culture Collection (ATCC, VA, USA), while the HEK293T cell line was provided by the Shanghai Institute of Cell Biology (Shanghai, China). The SKOV3, A2780, and OVCAR3 cell lines were continuously exposed to gradually increasing concentrations of cisplatin, resulting in the establishment of cisplatin-resistant lines (SKOV3-DDP, A2780-DDP, and OVCAR3-DDP). Successful development of these resistant lines was confirmed by a significant difference in the IC_{50} values compared to their parental cells. Cells were routinely cultured in high-glucose DMEM supplemented with 10% fetal bovine serum (FBS; Gibco, CA, USA) and 1% penicillin-streptomycin (New Cell & Molecular Biotech, Suzhou, China). Cultures were maintained at 37 °C in a 5% CO_2 atmosphere. All cell lines were authenticated by short tandem repeat (STR) analysis and verified to be free of mycoplasma contamination.

Immunohistochemistry

Hematoxylin and eosin (H&E) staining and immunohistochemistry (IHC) were conducted to compare organoids with their respective tumor tissues and to assess DNA damage and cell proliferation in tumor xenografts. The expression of biomarkers, including paired box gene 8 (PAX8), the proliferation marker Ki-67 (Ki67), and γ-H2A.X, was evaluated via IHC. Briefly, tumor tissue chips were deparaffinized in xylene and subsequently rehydrated in a graded ethanol series (100%, 95%, and 75%) followed by PBS, with each step lasting 5 minutes each. Antigen retrieval was performed by placing the slides in 0.01 M citrate buffer (pH 6.0) for 10-15 minutes in a boiling water bath. Following antigen retrieval, the tissues were blocked with normal goat serum for 10 minutes at room temperature and then incubated overnight at 4 °C with primary antibodies (γ-H2A.X, 1:500; Ki-67, 1:500). After washes in PBS, sections were incubated with either an HRP-conjugated secondary antibody or an Alexa Fluor 488-labeled secondary antibody for 1 hour at room temperature. Nuclei were counterstained with DAPI (Yeasen Biotechnology, Shanghai, China). Apoptosis was

detected using a TUNEL apoptosis detection kit (Beyotime, Shanghai, China) according to the manufacturer's instructions. Three random fields were imaged under a microscope, and the IHC and immunofluorescence (IF) results were visualized using a LEICA DMi8 microscope (Leica, Germany).

Luciferase report assay

The promoter sequence of LINC02776 was cloned into the pGL3.0-basic (Promega, Madison, WI, USA) Firefly luciferase reporter vector. Cells were transfected with these constructs using the Hieff Trans^{TM} Liposomal Transfection Reagent (Yeasen Biotechnology, Shanghai, China). The pRL-TK vector (Promega, Madison, WI, USA), encoding Renilla luciferase, was co-transfected as an internal control. Luciferase activities were quantified using the Dual-Luciferase Reporter Assay System (Promega, Madison, USA), following the manufacturer's protocol. The sequences of the PCR primers used for cloning are provided in Supplementary **Table S4**.

Cell proliferation and drug sensitivity assays

Cell proliferation was evaluated using the Cell Counting Kit-8 (CCK-8), 5-Ethynal-2'-deoxyuridine (EdU), and colony formation assays. For CCK-8 assays, OC cells were seeded at a density of 1×10^3 cells per well in 96-well plates and incubated overnight in DMEM containing 10% FBS. Next, 10 μL of CCK-8 reagent was added to each well, and the cells were incubated for an additional 2 hours at 37 °C. Absorbance at 450 nm was then measured to assess cell viability. For EdU assays, OC cells were seeded in 24-well plates and treated with or without cisplatin for 24 hours. They were then incubated with 10 μM EdU at 37 °C under 5% CO_2 for 2 hours. Afterward, the cells were stained with Hoechst 33342 (Beyotime, Shanghai, China) to visualize DNA. Fluorescence was measured to quantify cell proliferation. In colony formation assays, following transfection, OC cells were plated at a density of 1×10^3 cells per well in 6-well plates. After 2-3 weeks (depending on the growth rate of the specific cell line), the resulting colonies were fixed with 4% paraformaldehyde for 10 minutes and stained with crystal violet for 15 minutes. For drug sensitivity assays, OC cells were seeded at a density of 5×10^3 cells per well in 96-well plates for 24 hours and then treated with cisplatin or olaparib at the indicated concentrations for 48 hours. Subsequently, 10 μL of CCK-8

reagent was added to each well and incubated for 2 hours at 37 °C. Absorbance at 450 nm was measured to assess cell viability. Cisplatin and olaparib were purchased from MedChemExpress (Monmouth Junction, NJ, USA).

RNAScope, RNA fluorescence in situ hybridization (FISH), and immunofluorescence staining

For the RNAScope assay, a custom probe targeting LINC02776 (NR_187284.1) was synthesized by Advanced Cell Diagnostics. LINC02776 expression in OC cells was detected using the RNAscope 2.0 High Definition (HD) Assay kit (Advanced Cell Diagnostics, Minneapolis, MN, USA), following the manufacturer's protocol.

RNA FISH was performed using a FISH kit (RIBOBIO, Guangzhou, China) with minor modifications. Briefly, a Cy3-labeled LINC02776 probe was designed and synthesized by RiboBio (RIBOBIO, Guangzhou, China). OC cells were fixed with 4% formaldehyde at room temperature for 10 minutes, permeabilized with Triton X-100 (Sigma-Aldrich, China) for 10 minutes at room temperature, then washed three times with PBS. Cells were incubated overnight at 37 °C in the dark with the Cy3-labeled LINC02776 probes, and nuclei were counterstained with DAPI. Fluorescence images were acquired using a Leica TCS SP8 confocal microscope (Leica, Germany). For colocalization of LINC02776 and PARP1, cells were first incubated with the Cy3-labeled LINC02776 probe. An anti-PARP1 antibody was then applied overnight at 4 °C in the dark. For confocal imaging of phosphorylated histone H2AX (γ-H2A.X), BRCA1, and RAD51 foci, cells were cultured, treated with cisplatin, fixed, and permeabilized with Triton X-100. Primary antibodies were incubated overnight at 4 °C in the dark, followed by a fluorescence-labeled secondary antibody (Invitrogen, CA, USA) for one hour at room temperature in the dark. Cells were considered positive if they contained $\geq$ 10 foci of γ-H2A.X, BRCA1, or RAD51. Images were analyzed using Fiji software. A complete list of antibodies is provided in Supplementary **Table S7**.

RNA pull-down and RNA immunoprecipitation (RIP) assays

Biotinylated sense, antisense, or truncated LINC02776 RNAs were generated *in vitro* using the HiScribe^{TM} T7 High Yield RNA Synthesis Kit (New England Biolabs, Inc., USA). The PCR primers used for this procedure are listed in Supplementary **Table S8**.

Briefly, 4 μg of biotinylated LINC02776 RNA was heated in RNA folding buffer at 95 °C for 2 minutes, placed on ice for 5 minutes, and then incubated at room temperature for 30 minutes to promote secondary structure formation. The folded RNA was added to OC cell lysates and rotated overnight at 4 °C. Next, 40 μL of streptavidin beads (Thermo Fisher Scientific, IL, USA) was introduced into the RNA-protein mixture and incubated at room temperature for 1 hour with rotation. After washing, the beads were boiled in 1× SDS loading buffer. The retrieved proteins were analyzed by Western blotting and silver staining, and LINC02776-specific protein bands were identified by mass spectrometry. For the RNA immunoprecipitation (RIP) assay, PARP1 binding to LINC02776 was assessed using an RIP kit (Millipore, MA, USA). An IgG antibody served as the control.

5′ and 3′ RACE assays

To determine the specific transcript and full-length of LINC02776 in OC cells, 5' and 3' RACE (Rapid Amplification of cDNA Ends) were performed using the SMARTer™ RACE cDNA amplification kit (Clontech, Palo Alto, CA, USA) according to the manufacturer's instructions. The gene-specific PCR primers used for RACE are provided in Supplementary **Table S9**.

Caspase 3/7 assay and cell cycle analysis

Caspase 3/7 activity was assessed using the Caspase-Glo 3/7 Assay System (Promega, WI, USA) according to the manufacturer's instructions. In brief, 5×10^3 cells were seeded into each well of a 96-well plate and allowed to adhere overnight. The following day, cells were transfected with the indicated siRNAs. After 48 hours, the medium was removed, and luminescent substrates were added to each well in the dark. Luminescence was measured using a multimode plate reader. To evaluate caspase 3/7 activities in response to drug treatments, cells were treated with increasing concentrations of cisplatin for 24 hours prior to the assay.

Cells were seeded into six-well plates, serum-starved overnight to synchronize cell cycle, and then released from synchronization by adding complete medium. Twelve hours later, cells were harvested and fixed with 70% ethanol. Before flow cytometric analysis, the fixed cells were incubated with ribonuclease A (TaKaRa, Tokyo, Japan)

for 30 minutes at 37°C, followed by staining with propidium iodide (PI) (Sangon Biotech, Shanghai, China) for 30 minutes at room temperature.

HR, NHEJ, and A-NHEJ assays

U2OS cells containing the pEJ2-GFPpuro (#44025, Addgene), pimEJ5-GFPpuro (#44026, Addgene), and pDR-GFPpuro (#26475, Addgene) vectors were used to evaluate the efficiency of alternative non-homologous end joining (A-NHEJ), NHEJ, and HR efficiency, respectively. Cells were first infected with an I-SceI-containing lentivirus to include DNA double-strand breaks and subsequently transfected with the indicated plasmids or siRNA using jet-PRIME transfection reagent (Polyplus-transfection, Inc., New York, NY, USA). The percentage of GFP-positive cells, reflecting repair efficiency, was analyzed by flow cytometry using a 488 nm laser for excitation.

Chromatin immunoprecipitation

Chromatin immunoprecipitation (ChIP) assays were performed using the EZ-MagnaChIP HiSens Chromatin IP Kit (Merck Millipore, Darmstadt, Germany) following the manufacturer's protocol. Briefly, OC cells were washed twice with ice-cold PBS and cross-linked with 1% formaldehyde at 37 °C for 10 minutes. The reaction was quenched with glycine for 5 minutes. Cells were collected and sonicated using an Ultrasonic Cell Disruptor (Diagenode, Liège, Belgium) to generate 300-600 bp DNA fragments. After centrifugation, the supernatant containing chromatin fragments was collected and incubated with 40 μL Protein A/G magnetic beads (Thermo Fisher Scientific, IL, USA) overnight at 4°C. The chromatin was then immunoprecipitated with an HIF-1α antibody for 1 hour at room temperature with constant rotation. The DNA-protein complexes were washed with the provided washing buffer, and the target DNA fragments were extracted. The binding of the LINC02776 promoter to HIF-1α was analyzed using qRT-PCR. The sequences of the ChIP primers are listed in Supplementary **Table S10**.

PARP1 enzyme activity assay

To enzymatic activity of PARP1 was assessed *in vitro* using a commercial PARP1 activity assay kit (EMD Millipore, Billerica, MA, USA, 17-10149) following the

manufacturer's instructions. Biotin-labeled LINC02776 was synthesized *in vitro* via reverse transcription. A detection system was assembled, consisted of activated DNA, β-Nicotinamide adenine dinucleotide trihydrate (β-NAD), and recombinant PARP1 protein. The reaction mixture was incubated at room temperature for 30 minutes. Fluorescent was then measured using a fluorescent plate reader to determine PARP1 enzymatic activity.

Supplementary Tables

Supplementary Table S1. Clinical and pathologic characteristics of patients in the FUSCC1 cohort.

Parameter		NO of patients (n=110)
Age of surgery		
	≤60	79
	>60	31
Grade (FIGO)		
	II	7
	III	83
	IV	20
Lymph node metastasis		
	Yes	30
	No	80
R0		
	Yes	67
	No	43
Histological subtype		
	HGSOC	89
	LGSOC	4
	MC	3
	EC	4
	CCC	10
Chemoregimen		
	TC	102
	CBP+CTX	4
	DP	2
	TP	2

Abbreviations: HGSOC, High grade serous carcinoma; LGSOC: Low grade serous carcinoma; MC: Mucinous carcinoma; EC, Endometrioid carcinoma; CCC: Clear cell carcinoma; TC, Paclitaxel+Carboplatin; CBP+CTX, Carboplatin+Cyclophosphamide; DP, Docetaxel+Cisplatin; TP, Paclitaxel+Cisplatin.

Supplementary Table S2. Clinical and pathological characteristics of patients whose samples were used for RNA sequencing.

Number	Age	Grade (FIGO)	Residual disease	Histological subtype	PFI (month)	Platinum status	Chemoregimen
Sample 1	49	IIIC	R0	HGSOC	16.2	Sensitive	TC
Sample 2	46	IIIC	R0	HGSOC	33.8	Sensitive	TC
Sample 3	50	IIIB	R0	HGSOC	15.9	Sensitive	TC
Sample 4	56	IIIC	R0	HGSOC	33.4	Sensitive	TC
Sample 5	64	IIIC	R0	HGSOC	33.3	Sensitive	TC
Sample 6	55	IIIC	R0	HGSOC	32.9	Sensitive	TC
Sample 7	49	IIIB	R0	HGSOC	21.3	Sensitive	TC
Sample 8	50	IIIC	R0	HGSOC	40.1	Sensitive	TC
Sample 9	36	IVB	R0	HGSOC	32.9	Sensitive	TC
Sample 10	44	IVB	R0	HGSOC	20.3	Sensitive	TC
Sample 11	64	IIIC	R0	HGSOC	1.3	Resistant	TC
Sample 12	49	IIIC	R0	HGSOC	3.7	Resistant	TC
Sample 13	43	IIIC	R0	HGSOC	3.9	Resistant	TC
Sample 14	52	IVB	R0	HGSOC	2.4	Resistant	TC
Sample 15	43	IIIC	R0	HGSOC	4.2	Resistant	TC
Sample 16	55	IIIC	R0	HGSOC	1.4	Resistant	TC
Sample 17	66	IIIC	R0	HGSOC	3.2	Resistant	TC
Sample 18	43	IIIC	R0	HGSOC	3.9	Resistant	TC
Sample 19	57	IVB	R0	HGSOC	4.5	Resistant	TC

Supplementary Table S3. Differentially expressed lncRNAs between tissues from platinum-resistant and -sensitive patients with ovarian cancer.

Supplementary Table S4. SiRNAs, shRNAs and primer sequences used in this study.

siRNAs sequences used in this study

	Sequences
LINC02776-siRNA1	GCGAGAGTTCTGGATCTCATC
LINC02776-siRNA2	GATGGCGAGAGTTCTGGAT
HIF-1α-siRNA1	GAAGGAACCUGAUGCUUUA
HIF-1α-siRNA2	CUGAUGACCAGCAACUUGA
HIF-1α-siRNA3	CAAUCAAGAAGUUGCAUUA
AL391335.1-siRNA1	GCTGATGGAGACGCTAGATTCCAAA
AL391335.1-siRNA2	CCGAGAAGAGAAACTACGGTGTCAA
AC010761.1-siRNA1	CCAATCACTTTGTTCAGACTTTGGT
AC010761.1-siRNA2	CAGGCCATAACTGCCAAATACAGAT
AC097359.2-siRNA1	CACCTGCAATCATCCTACAAGATCT
AC097359.2-siRNA2	GCAAGCCAGGGAAGCTTAATCTGTA

shRNAs sequences used in this study

	Forward primer (5'-3')	Reverse primer (5'-3')
LINC02776-shRNA1	CACCGCGAGAGTTCTGGATCTCATCTCAAGGATGAGATCCAGAACTCTCGCTTTTTT	AAACAAAAAAGCGAGAGTTCTGGATCTCATCCTTGAGATGAGATCCAGAACTCTCGC
LINC02776-shRNA2	CACCGATGGCGAGAGTTCTGGATTCAAGATCCAGAACTCTCGCCATCTTTTTT	AAACAAAAAAGATGGCGAGAGTTCTGGATCTTGAATCCAGAACTCTCGCCATC
PARP1-shRNA1	CACCGCAAAGGCCAGGATGGAATTGTCAAGCAATTCCATCCTGGCCTTTGCTTTTTT	AAACAAAAAAGCAAAGGCCAGGATGGAATTGCTTGACAATTCCATCCTGGCCTTTGC
PARP1-shRNA2	CACCGGACCAAGTGTATGGTCAAGATCAAGTCTTGACCATACACTTGGTCCTTTTTT	AAACAAAAAAGGACCAAGTGTATGGTCAAGACTTGATCTTGACCATACACTTGGTCC

Primers for cloning

	Forward primer (5'-3')	Reverse primer (5'-3')
PCDH-LINC02776	AGAAGATTCTAGAGCTAGCGGGAGACCGGCGACGCTCTCC	CTGCAGCTCGAGCCCGGGGGTAAATCTTTTAAAGAGTCGCAAGGTT
PCDH-PARP1-3Xflag-FL	TGACGATGACAAGGAATTCATGGCGGAGTCTTCGGATAA	GCTCGAGCCCGGGGGATCCTTACCACAGGGAGGTCTTAA
PCDH-PARP1-3xflag-A	TGACGATGACAAGGAATTCATGGCGGAGTCTTCGGATAA	GCTCGAGCCCGGGGGATCCCCTGAGCAGACTGTAGGCCA
PCDH-PARP1-3xflag-B	TGACGATGACAAGGAATTCATGGCGGAGTCTTCGGATAA	GCTCGAGCCCGGGGGATCCGTGCGCTAAGAACAACTCCT

PCDH-PARP1-3xflag-C	TGACGATGACAAGGAATTCATGGCGGAGTCTTCGGATAA	GCTCGAGCCCGGGGGATCCCTTGACTCCTGGGAGCTGCT
PCDH-PARP1-3xflag-D	TGACGATGACAAGGAATTCAGTGAAGGAAAGAGAAAAGG	GCTCGAGCCCGGGGGATCCTTACCACAGGGAGGTCTTAA
LINC02776-promoter-PGL3(-150~+950)	ATTTCTCTATCGATAGGTACCGTCCCCTTCCTCTTCCTCA	CCAACAGTACCGGAATGCCAGGTCTTCTAAACAGAAGCTG
LINC02776-promoter-PGL3(-150~+30)	ATTTCTCTATCGATAGGTACCGTCCCCTTCCTCTTCCTCA	CCAACAGTACCGGAATGCCAGCTTTGCTCCCGCGCCCGCC
LINC02776-promoter-PGL3(+30~+560)	ATTTCTCTATCGATAGGTACTCTGAGTCACCGGCCACCAA	CCAACAGTACCGGAATGCCAAGGGTTTGGCTAAAATGAATTGTC
LINC02776-promoter-PGL3(+560~+950)	ATTTCTCTATCGATAGGTACCGCTTTTGAGAAGGGGAAGA	CCAACAGTACCGGAATGCCAGGTCTTCTAAACAGAAGCTG

Supplementary Table S5. Clinical characteristics of ovarian cancer PDOs.

Patient ID	Age (years)	Histological subtype	Sample from HGSOC	Platinum-free interval, PFI (months)	Estimated PARPi duration （months）
Patient 1	49	HGSOC	Ascites	3	-
Patient 2	80	HGSOC	Colonic mesentery	-	Olaparib 10

Supplementary Table S6. RNA-seq data of LINC02776 knockdown A2780 cells and the GO enriched genes list.

Supplementary Table S7. Antibodies used in this study.

ANTIBODIES	SOURCE	IDENTIFIER
GAPDH	Proteintech	Cat.60004-1-Ig
β-actin	Proteintech	Cat.60008-1-Ig
PAX8	Proteintech	Cat.10336-1-AP
Ki67	Proteintech	Cat.27309-1-AP
γ-H2A.X	Cell Signaling Technology	Cat.9718
H2A.X	Cell Signaling Technology	Cat.7631
RAD51	Cell Signaling Technology	Cat.65653
BRCA1	Cell Signaling Technology	Cat.50799
PARP1	Proteintech	Cat.13371-1-AP
PAR	R&D Systems	Cat.4335-MC-100
SF3B3	Proteintech	Cat.14577-1-AP
SF3B1	Proteintech	Cat.27684-1-AP
DDX46	Proteintech	Cat.16927-1-AP
EFTUD2	Proteintech	Cat.10208-1-AP
FLAG	Sigma–Aldrich	Cat.F3040
HIF-1α	Cell Signaling Technology	Cat.36169

Supplementary Table S8. Primers for RNA pulldown used in this study.

	Forward primer (5'-3')	Reverse primer (5'-3')
LINC02776-(1-335nt)-Pulldown-sense	GATCACTAATACGACTCACTATAGGGGAGACCGGCGACGCTCTCC	TTTTAAAGAGTCGCAAGGTT
LINC02776-(1-90nt)-Pulldown-sense	GATCACTAATACGACTCACTATAGGGGAGACCGGCGACGCTCTCC	GTGCGGGAGGGCAGACCGGA
LINC02776-(91-220nt)-Pulldown-sense	GATCACTAATACGACTCACTATAGGCGGCGCCTTTGGGCTCAGCG	CGTGTCCGGGCCTTTTGGGC
LINC02776-(221-335nt)-Pulldown-sense	GATCACTAATACGACTCACTATAGGCTACTCCAAAACGTCTCTCC	TTTTAAAGAGTCGCAAGGTT
LINC02776-(335-1nt)-Pulldown-antisense	GGAGACCGGCGACGCTCTCC	GATCACTAATACGACTCACTATAGGTTTTAAAGAGTCGCAAGGTT

Supplementary Table S9. Primers for RACE assays used in this study.

	Sequences
LINC02776-RACE-5'GSP	TCTCGCCATCTGGCCACTGG
LINC02776-RACE-3'GSP	CCAGTGGCCAGATGGCGAGA

Supplementary Table S10. Primers for ChIP-qPCR, qRT-PCR and semi-qRT-PCR used in this study.

Primers for ChIP-qPCR

	Forward primer (5'-3')	Reverse primer (5'-3')
ChIP negative-qPCR	AGAAATGAAAAAGCACCTCCCC	TACTGCCTTGGAGAAGCCTG
HRE1-qPCR	GTCCCTCGGTCCGGTCTGCC	CCCCAATTTACTCTCGCCAT
HRE2-qPCR	CAGGTTCTGGATCTCATCAG	ACGTTTTGGAGTAGCGTGTC
HRE3-qPCR	AAAAGGCCCGGACACGCTAC	TCTTTTCCTGTCGTCCCCAA
HRE4-qPCR	AAATCTAACCTTGCGACTCT	AATTGTCTGAAGGGACCTCC

Primers for qRT-PCR

	Forward primer (5'-3')	Reverse primer (5'-3')
LINC02776-qPCR	GGCGAGAGTTCTGGATCTCA	GGTTAGATTTCAGGAGCCCT
LINC02776-isoform2-qPCR	AGGAAAAGATATGGCCCATA	AATTGTCTGAAGGGACCTCC
AL391335.1-qPCR	CGGTGTCAAATCCCACGCCC	GGGTCTGGGCTCCACTGCGC
AC010761.1-qPCR	AAGGGCTCTTACAACCTCAA	TGTGACTTTAAATGGGGAAA
AC097359.2-qPCR	GCGACGGCTACGGCGACTTCTTTAC	CAGCCAGTGGCACCCGGAAG
β-actin-qPCR	TTGTTACAGGAAGTCCCTTGCC	ATGCTATCACCTCCCCTGTGTG
PARP1-qPCR	CCAAGCCAGTTCAGGACCTCAT	GGATCTGCCTTTTGCTCAGCTTC
U2-qPCR	ATCGCTTCTCGGCCTTTTGG	AGGTCGATGCGTGGAGTGGA
lncMER52A-qPCR	GCCAAATACAGCCTGCTGGG	CCACTGCGAAGATCAAACCG
lncLUCAT1-qPCR	TTGGCACCAGAGACCACAAA	GGGCGACAGAGCGAAACTCT
RC3H1-qPCR	TCCACAATGGACGGATTTCCT	AACCCAAACTGATGGGCTTTC
RC3H1-IT1-qPCR	CCAGAGTGCTGGGATTACAG	CCCACTTCTGTATAGGCATT

Primers for semi-qRT-PCR

	Forward primer (5'-3')	Reverse primer (5'-3')
LINC02776-semi-qPCR	ACCGGCGCCTTTGGGCTCAG	GGGTGACGAGGACAAAGCTG

Supplementary Figures

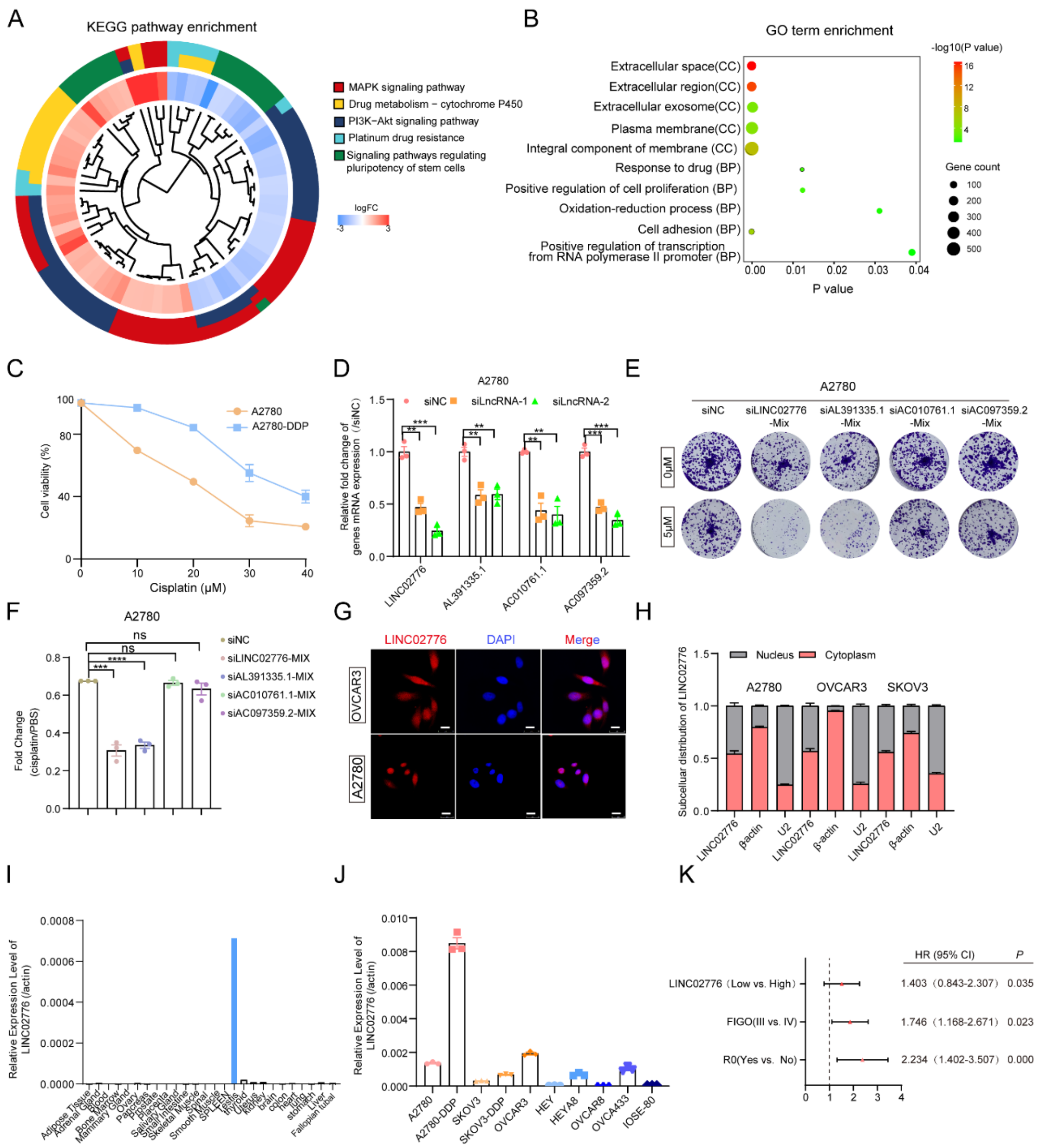

Supplementary Figure S1. Upregulation of LINC02776 in platinum-resistant OC. (**A, B**) Top KEGG pathways (A) and Gene Ontology (GO) biological processes (B) enriched by differentially expressed coding genes ($P < 0.05$, $|\log2(FC)| > 1$). (**C**) Cisplatin sensitivity assay (CCK-8) comparing A2780 cells ($IC_{50} = 20.13$ μM) with A2780-DDP cells ($IC_{50} = 32.25$ μM), highlighting increased resistance in A2780-DDP cells. (**D**) qRT-PCR analysis showing the knockdown efficiency of indicated siRNAs transfected into A2780 cells. (**E**) Colony formation assays demonstrating changes in cell number following cisplatin treatment at various concentrations over two weeks in A2780 cells transfected with indicated siRNAs. Colonies were visualized using crystal violet staining. (**F**) Proliferation fold change induced by cisplatin was quantified in siLINC02776-transfected cells relative to siNC controls. (**G**) RNA FISH analysis showing the cellular distribution of LINC02776 in A2780 and OVCAR3 cells. Cy3-

labeled probes (red) were used to detect LINC02776, and DAPI (blue) was used to stain nuclei. Scale bar: 20 μm. (**H**) Intracellular localization of LINC02776 was determined by qRT-PCR, using β-actin mRNA (cytoplasmic marker) and U2 snRNA (nuclear marker) as controls. (**I**) Expression levels of LINC02776 in human normal tissues were examined. (**J**) qRT-PCR analysis showing LINC02776 expression levels across various OC cell lines. (**K**) Multivariate survival analysis indicating that LINC02776 serves as an independent prognostic factor for OC patients. Values are presented as mean ± SEM, n = 3 for panels (C-I). Statistical significance is indicated as follows: ns, not significant; *$P < 0.05$; **$P < 0.01$; ***$P < 0.001$; ****$P < 0.0001$.

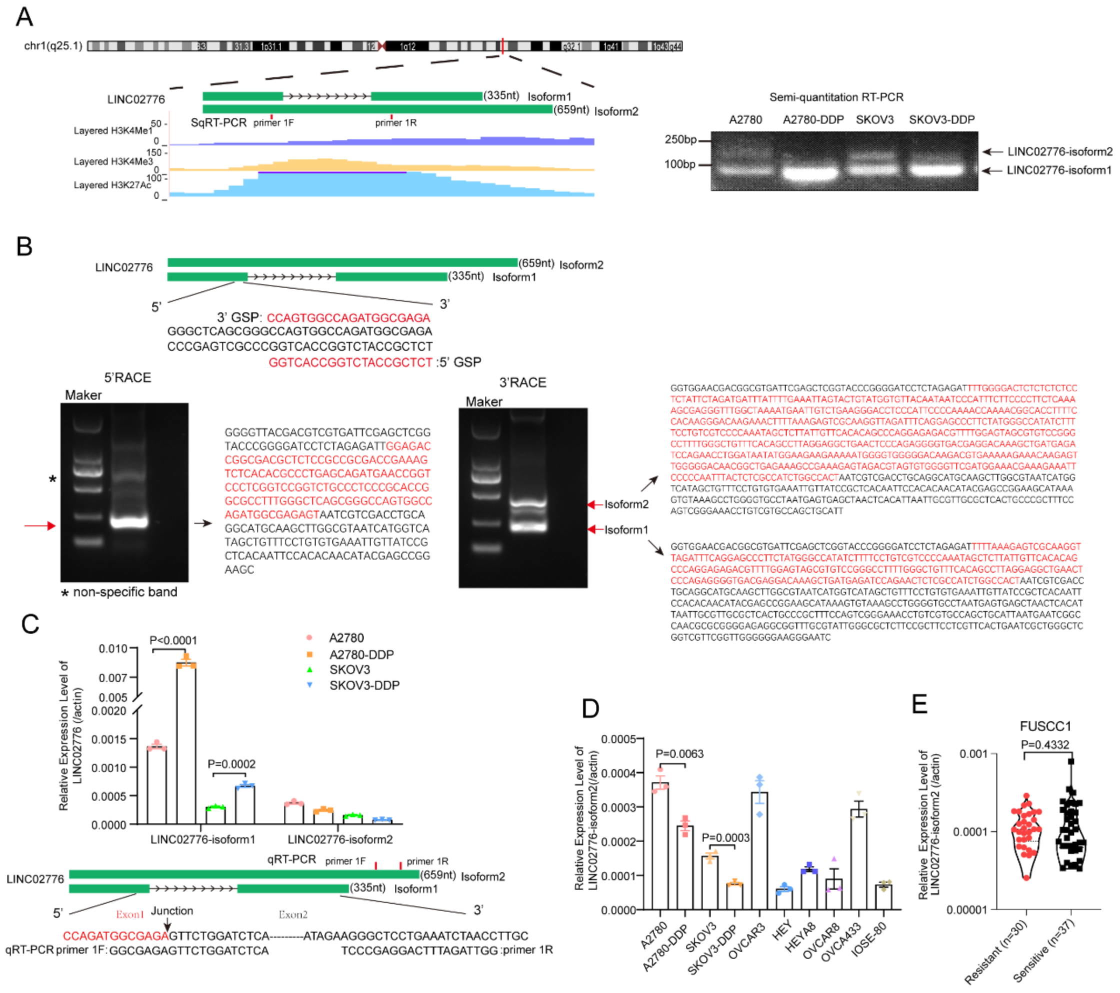

Supplementary Figure S2. Characteristics of LINC02776. (**A**) Left: UCSC Genome Browser view showing the genomic characteristics of LINC02776. Right: Semi-quantitative RT-PCR analysis detecting mRNA expression levels of LINC02776-isoform1 and LINC02776-isoform2 in OC cells. (**B**) Top: The positions of 5′ and 3′ RACE primers are indicated within the LINC02776 sequence. Bottom: Representative agarose gel electrophoresis images and Sanger sequencing results for PCR products generated by 5′-RACE (left panel) and 3′-RACE (right panel). (**C**) qRT-PCR analysis showing LINC02776 isoform expression levels in A2780-DDP and SKOV3-DDP cells and their parental lines. The primer design for detecting LINC02776-isoform1 and LINC02776-isoform2 is illustrated below. (**D**) qRT-PCR quantification showing LINC02776-isoform2 expression levels across various OC cell lines. (**E**) Comparative analysis of LINC02776-isoform2 expression between platinum-resistant and platinum-sensitive OC tissues. Values are presented as mean ± SEM, n = 3 for panels (B-E), *P < 0.05; **P < 0.01; ***P < 0.001; ****P < 0.0001.

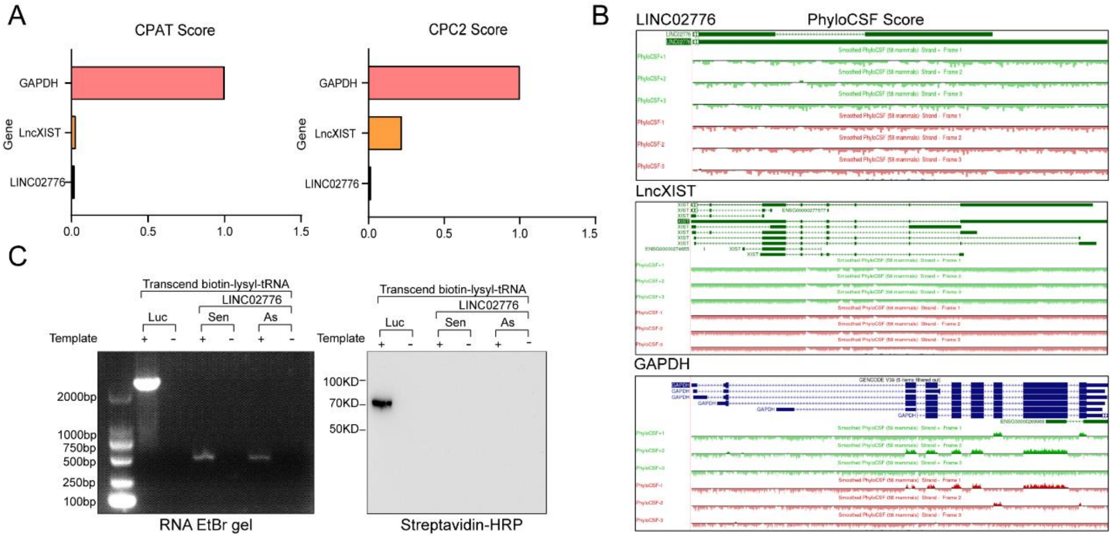

Supplementary Figure S3. LINC02776 lacks protein-coding potential. (**A, B**) The protein-coding potential of LINC02776 was evaluated using three computational tools: Coding Potential Assessment Tool (CPAT, left panel in A), Coding Potential Calculator (CPC, http://cpc.gao-lab.org, right panel in A), and PhyloCSF software (**B**). GAPDH served as a positive control for coding genes, while lncXIST served as a negative control for non-coding genes. (**C**) *In vitro* transcription and translation assays were performed on both the sense and antisense transcripts of LINC02776. Luciferase (Luc) was used as a positive control for translation. Values are presented as mean ± SEM (n = 3).

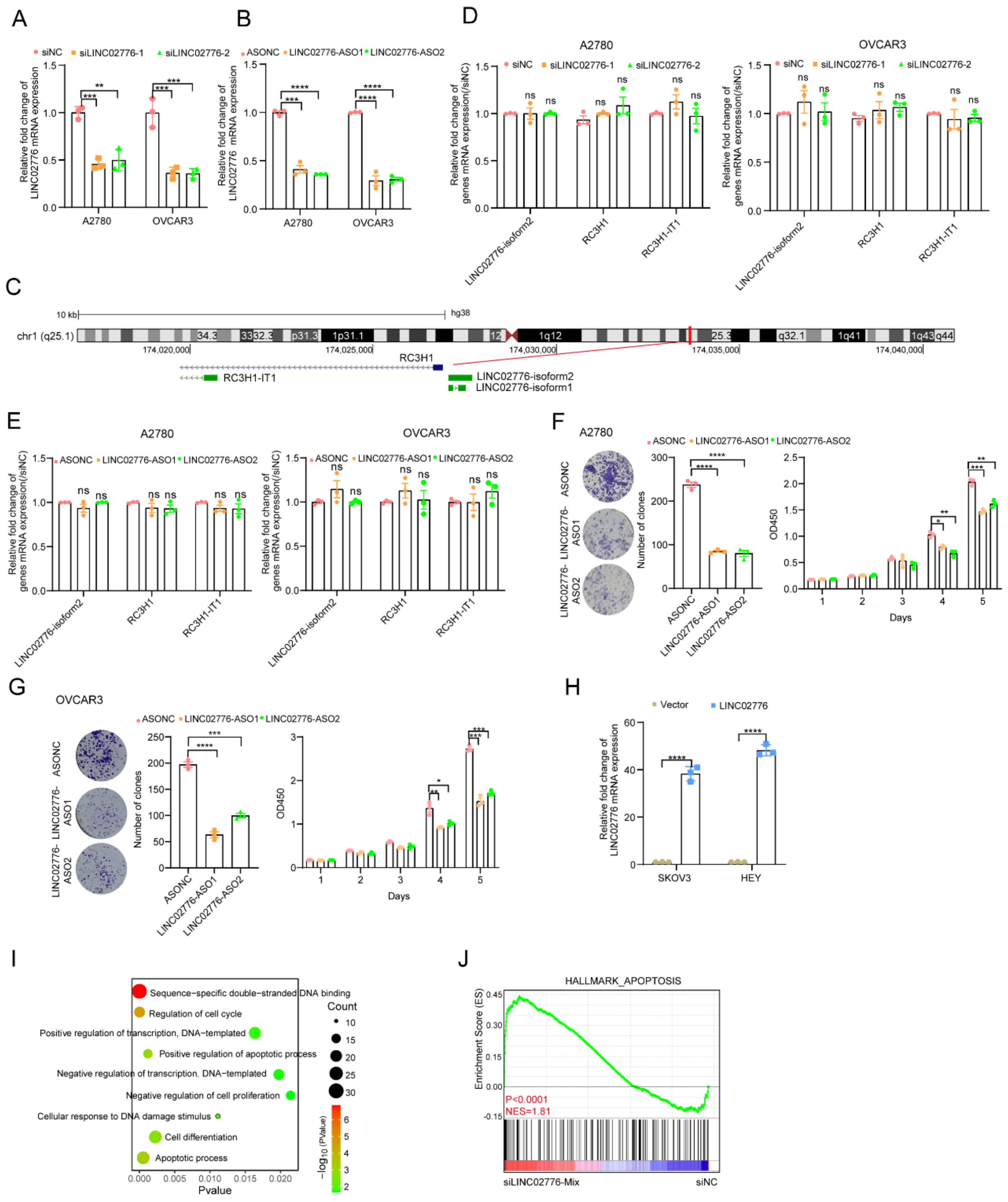

Supplementary Figure S4. LINC02776 regulates DNA damage and apoptosis in OC cells. (**A**) qRT-PCR analysis showing LINC02776 mRNA levels in OC cells transfected with two independent siRNAs targeting LINC02776. (**B**) qRT-PCR analysis showing LINC02776 mRNA levels in OC cells transfected with two independent ASOs targeting LINC02776. (**C**) Schematic representation of the LINC02776 genomic region and its neighboring genes based on the UCSC Genome Browser. (**D**) qRT-PCR analysis showing mRNA levels of LINC02776-isoform2, RC3H1, and RC3H1-IT1 in OC cells transfected with two independent siRNAs targeting LINC02776-isoform1. (**E**) qRT-PCR analysis showing mRNA levels of LINC02776-isoform1, LINC02776-isoform2, RC3H1, and RC3H1-IT1 in OC cells transfected with two independent ASOs targeting LINC02776-isoform1. (**F, G**) Colony formation assays and CCK-8 assays showing cell proliferation and survival in A2780 (F) and OVCAR3 (G) cells transfected with

LINC02776 ASOs, with representative images provided. (**H**) qRT-PCR analysis showing LINC02776 mRNA levels in OC cells transfected with either an empty vector or the PCDH-LINC02776 plasmid. (**I**) GO analysis showing the top nine biological processes regulated by LINC02776. (**J**) Gene Set Enrichment Analysis (GSEA) demonstrating a negative correlation between LINC02776 mRNA expression and apoptosis signatures based on RNA-seq data. Values are presented as mean ± SEM, n = 3 for panels (A-H). Statistical significance is indicated as follows: ns, not significant; **P* < 0.05; ***P* < 0.01; ****P* < 0.001; *****P* < 0.0001.

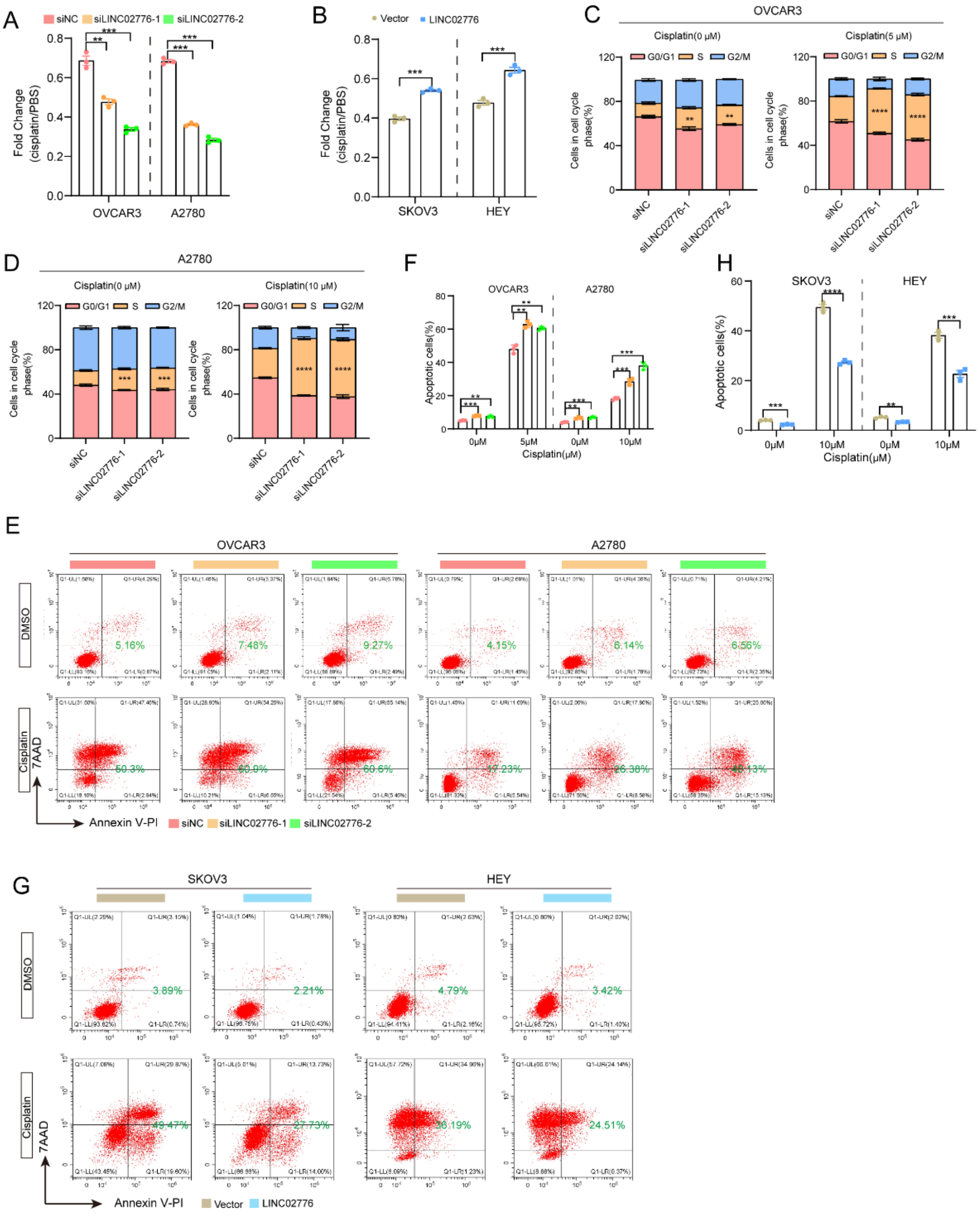

Supplementary Figure S5. LINC02776 regulates apoptosis in OC cells. (**A**) Quantification of proliferation fold change induced by cisplatin in siLINC02776-transfected OVCAR3 and A2780 cells, compared to siNC-transfected controls. (**B**) Quantification of proliferation fold change induced by cisplatin in PCDH-LINC02776-transfected SKOV3 and HEY cells, compared to vector-transfected controls. (**C, D**) Cell cycle profiles of untreated or cisplatin-treated OVCAR3 (C) and A2780 (D) cells, transfected with siNC or siLINC02776, analyzed vi flow cytometry 48 hours post-treatment. (**E, F**) Representative images (E) and quantification (F) of apoptosis analysis in OVCAR3 and A2780 cells, transfected with siNC or siLINC02776, following 48 hours of cisplatin treatment. (**G, H**) Representative images (G) and quantification (H) of apoptosis analysis in SKOV3 and HEY cells, transfected with vector or PCDH-LINC02776, following 48 hours of cisplatin treatment. Values are presented as mean ± SEM, n = 3 for all panels. Statistical significance is indicated as follows: *$P < 0.05$; **$P < 0.01$; ***$P < 0.001$; ****$P < 0.0001$.

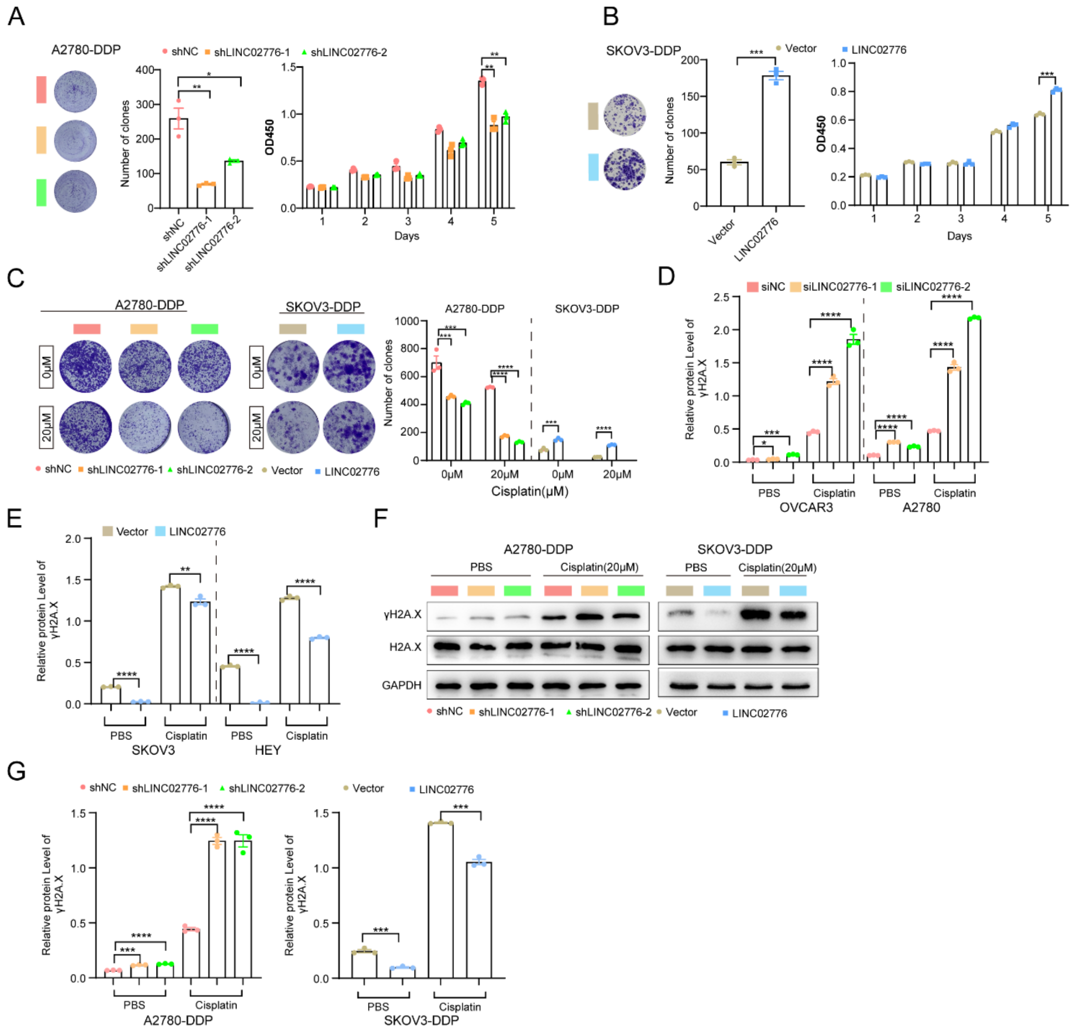

Supplementary Figure S6. Knockdown of LINC02776 enhances cisplatin sensitivity in cisplatin-resistant OC cells. (**A**) Left: Representative images and quantification of colony formation assays in A2780-DDP cells stably depleted of LINC02776 via shRNA. Right: CCK-8 assay results showing cell viability in A2780-DDP cells stably depleted of LINC02776 via shRNA. (**B**) Left: Representative images and quantification of colony formation assays in SKOV3-DDP cells stably overexpressing PCDH-LINC02776. Right: CCK-8 assay results showing cell viability in SKOV3-DDP cells stably overexpressing PCDH-LINC0276. (**C**) Colony formation assays with representative images and quantification in A2780-DDP and SKOV3-DDP cells transfected with shLINC02776 or PCDH-LINC02776, showing changes in cell number in response to cisplatin treatment at various concentrations over two weeks. Colonies were visualized using crystal violet staining. (**D**) Quantification of γ-H2A.X protein levels corresponding to data presented in Figure 3E. (**E**) Quantification of γ-H2A.X protein levels corresponding to data presented in Figure 3F. (**F**) Western blot analysis showing γ-H2A.X levels in cisplatin-resistant OC cells treated with 20 μM cisplatin for 24 hours. (**G**) Quantification of γ-H2A.X protein levels corresponding to data presented in panel (F). Values are expressed as mean ± SEM, n = 3 for all panels (A-G). Statistical significance is indicated as follows: **P* < 0.05; ***P* < 0.01; ****P* < 0.001; *****P* < 0.0001.

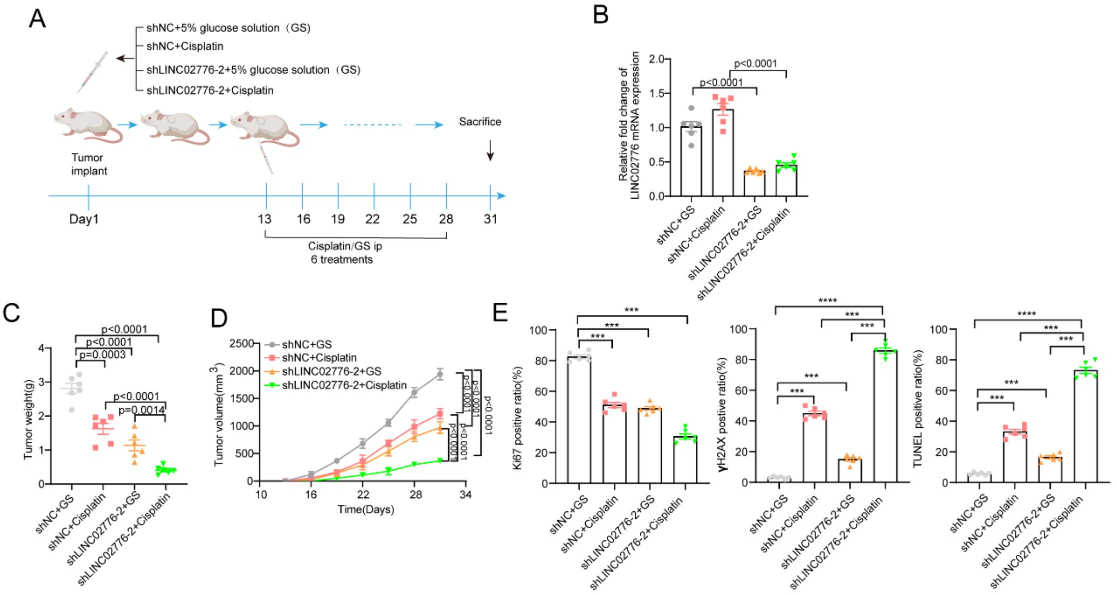

Supplementary Figure S7. Knockdown of LINC02776 enhances cisplatin sensitivity in OC cells *in vivo*. (**A**) Schematic representation of the *in vivo* experimental procedure, illustrating treatments including LINC02776 knockdown alone or in combination with cisplatin in a xenograft mouse model. (**B**) qRT-PCR analysis showing LINC02776 RNA levels in xenograft tumors harvested from treated mice. (**C**) Tumor weight assessment in xenograft mice subjected to the indicated treatments. (**D**) Tumor volume measurement in xenograft mice subjected to the indicated treatments. n = 6 tumors per group, analyzed using a two-sided Student's *t*-test. (**E**) Quantification of Ki-67-positive cells, γ-H2A.X-positive cells, and TUNEL-positive cells, corresponding to the data presented in Figure 3L, assessing cell proliferation, DNA damage, and apoptosis. Values are expressed as mean ± SEM. Statistical significance is indicated as follows: **P* < 0.05; ***P* < 0.01; ****P* < 0.001; *****P* < 0.0001.

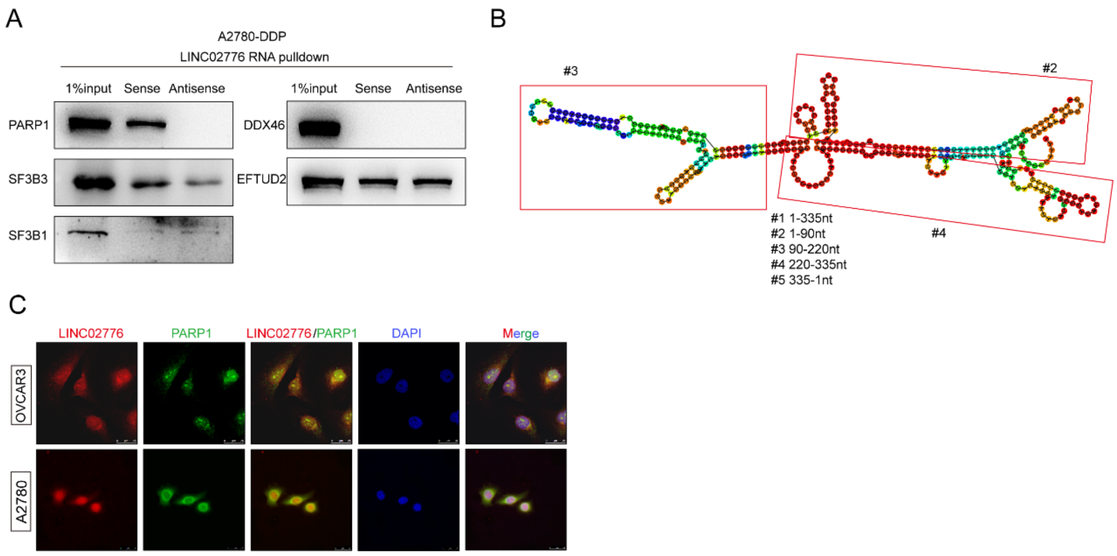

Supplementary Figure S8. Identification of LINC02776-interacting proteins. (**A**) Western blot analysis showing the interaction between PARP1 and biotinylated LINC02776 in A2780-DDP cells. (**B**) Predicted secondary structure of LINC02776, generated using RNAfold software (http://rna.tbi.univie.ac.at/cgi-bin/RNAWebSuite/RNAfold.cgi). The deletion mapping of biotinylated LINC02776 RNA motifs is illustrated, indicating key regions involved in PARP1 binding. (**C**) Colocalization analysis showing LINC02776 and PARP1 in OC cells using fluorescence microscopy. Scale bar: as indicated in the respective panels.

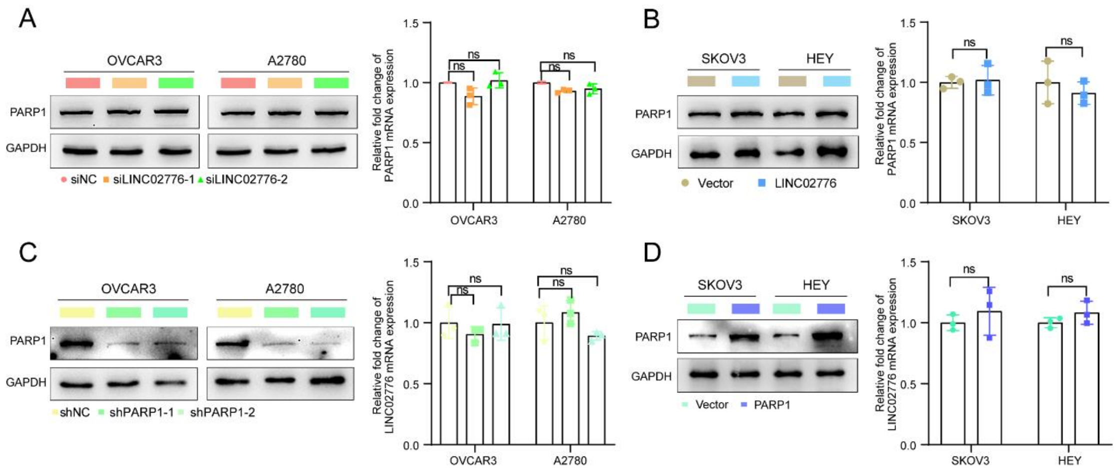

Supplementary Figure S9. LINC02776 and PARP1 do not influence each other's mRNA or protein expression levels. (**A**) Western blot and qRT-PCR analyses showing PARP1 protein and mRNA expression levels in OC cells following LINC02776 knockdown. (**B**) Western blot and qRT-PCR analyses showing PARP1 protein and mRNA expression levels in OC cells with LINC02776 overexpression. (**C**) qRT-PCR analysis showing LINC02776 mRNA expression levels in OC cells following PARP1 knockdown. (**D**) qRT-PCR analysis showing LINC02776 mRNA expression levels in OC cells with PARP1 overexpression. Values are presented as mean ± SEM from three independent experiments (n = 3). Statistical significance is indicated as follows: ns, not significant; *P < 0.05; **P < 0.01; ***P < 0.001; ****P < 0.0001.

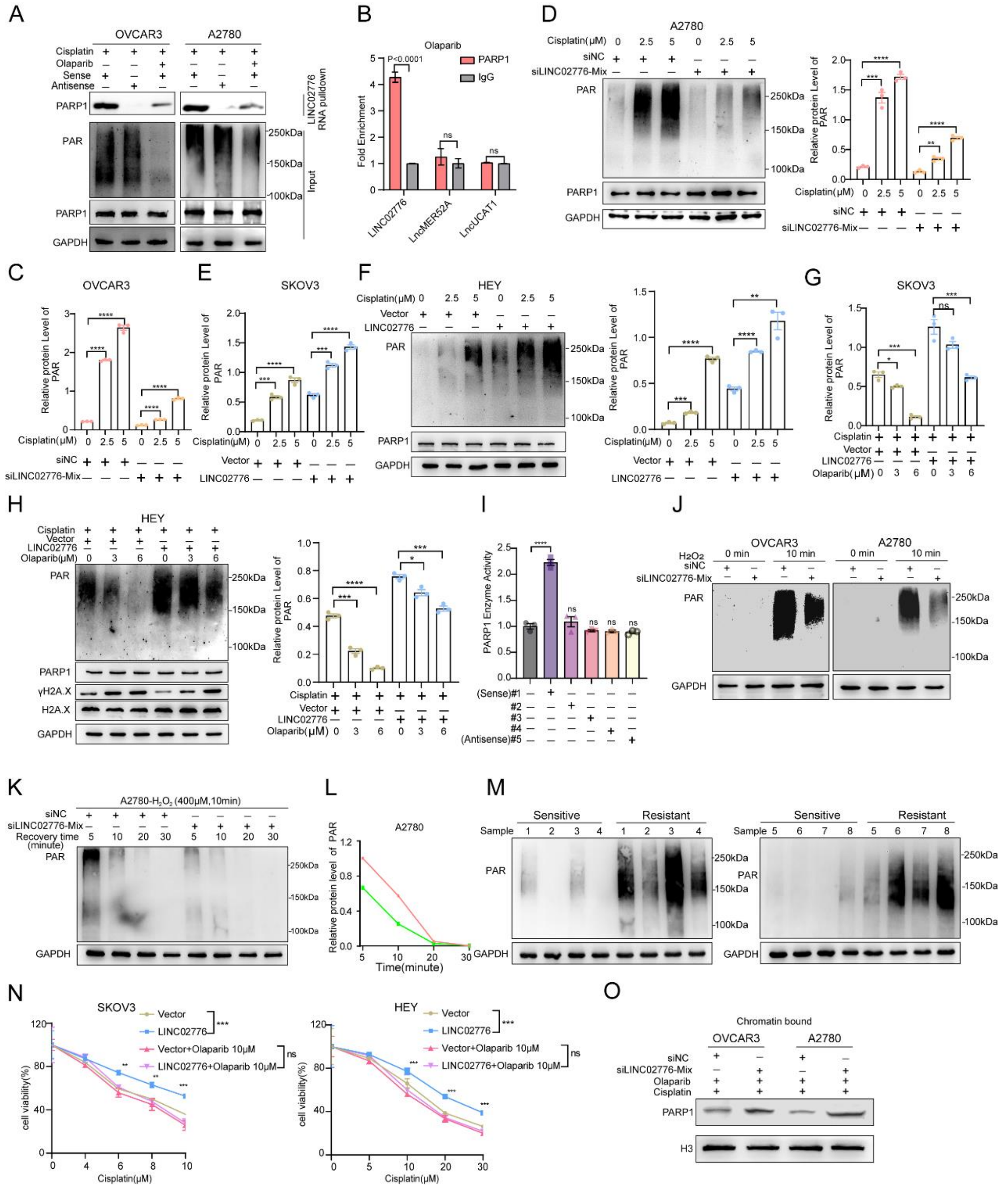

Supplementary Figure S10. LINC02776 enhances DNA damage-triggered PARylation in OC cells. (**A**) Western blot analysis showing PARP1 proteins precipitated using biotinylated LINC02776 RNA probes (sense and antisense) in OVCAR3 and A2780 cells treated with cisplatin (OVCAR3: 2.5 μM; A2780: 5 μM) for 24 hours followed by olaparib (6 μM) for 4 hours. The levels of PAR, PARP1, and GAPDH were detected in input samples. (**B**) RIP assays using a PARP1 antibody were performed on A2780 cell lysates treated with olaparib (6 μM) for 4 hours. Co-immunoprecipitated RNA was analyzed by qRT-PCR for LINC02776 expression, with LncMER52A and LncUCAT1 serving as negative controls. (**C**) Quantification of PAR protein levels corresponding to data from Figure 4I. (**D**) Left: Western blot analysis of PAR, PARP1, and GAPDH protein levels in A2780 cells transfected with siNC or LINC02776-specific siRNA, treated with cisplatin (2.5 μM or 5 μM) for 24 hours. Right:

Quantitative analysis of PAR protein levels. (**E**) Quantification of PAR protein levels corresponding to data from Figure 4J. (**F**) Left: Western blot analysis of PAR, PARP1, and GAPDH protein levels in HEY cells transfected with vector control or PCDH-LINC02776, treated with cisplatin (2.5 μM or 5 μM) for 24 hours. Right: Quantitative analysis of PAR protein levels. (**G**) Quantification of PAR protein levels corresponding to data from Figure 4K. (**H**) Left: Western blot analysis of PAR, PARP1, γ-H2A.X, H2A.X, and GAPDH in HEY cells overexpressing LINC02776, treated with various concentrations of olaparib for 4 hours, followed by 5 μM cisplatin for 24 hours. Right: Quantitative analysis of PAR protein levels. (**I**) Activity assessment of human recombinant PARP1 in response to different truncations of LINC02776. (**J**) Western blot analysis showing PAR protein levels in OVCAR3 and A2780 cells treated with 400 μM H_2O_2 for specified durations (0 and 10 minutes) after LINC02776 depletion. (**K**) Western blot analysis showing PAR protein levels in A2780 cells transfected with LINC02776 siRNA, treated with 400 μM H_2O_2 for 10 minutes, followed by replacement with normal culture medium. Lysates were collected at 5, 10, 20, and 30 minutes post-treatment. (**L**) Quantitative analysis of PAR protein levels corresponding to data from panel (K). (**M**) Comparative analysis of PAR protein levels in platinum-resistant versus platinum-sensitive OC tissues using western blot analysis. (**N**) Cell viability assays showing IC_{50} values in vector control and LINC02776-overexpressing SKOV3 and HEY cells treated with cisplatin (48 hours) with or without 10 μM olaparib. (**O**) Western blot analysis of chromatin-bound PARP1 protein levels in OVCAR3 and A2780 cells transfected with siNC or siLINC02776-MIX, pre-treated with 3 μM olaparib (4 hours) followed by cisplatin (OVCAR3: 2.5 μM; A2780: 5 μM) for 24 hours. Values are presented as mean ± SEM, n = 3 for panels (A-H). Statistical significance is indicated as follows: ns, not significant; **P* < 0.05; ***P* < 0.01; ****P* < 0.001; *****P* < 0.0001.

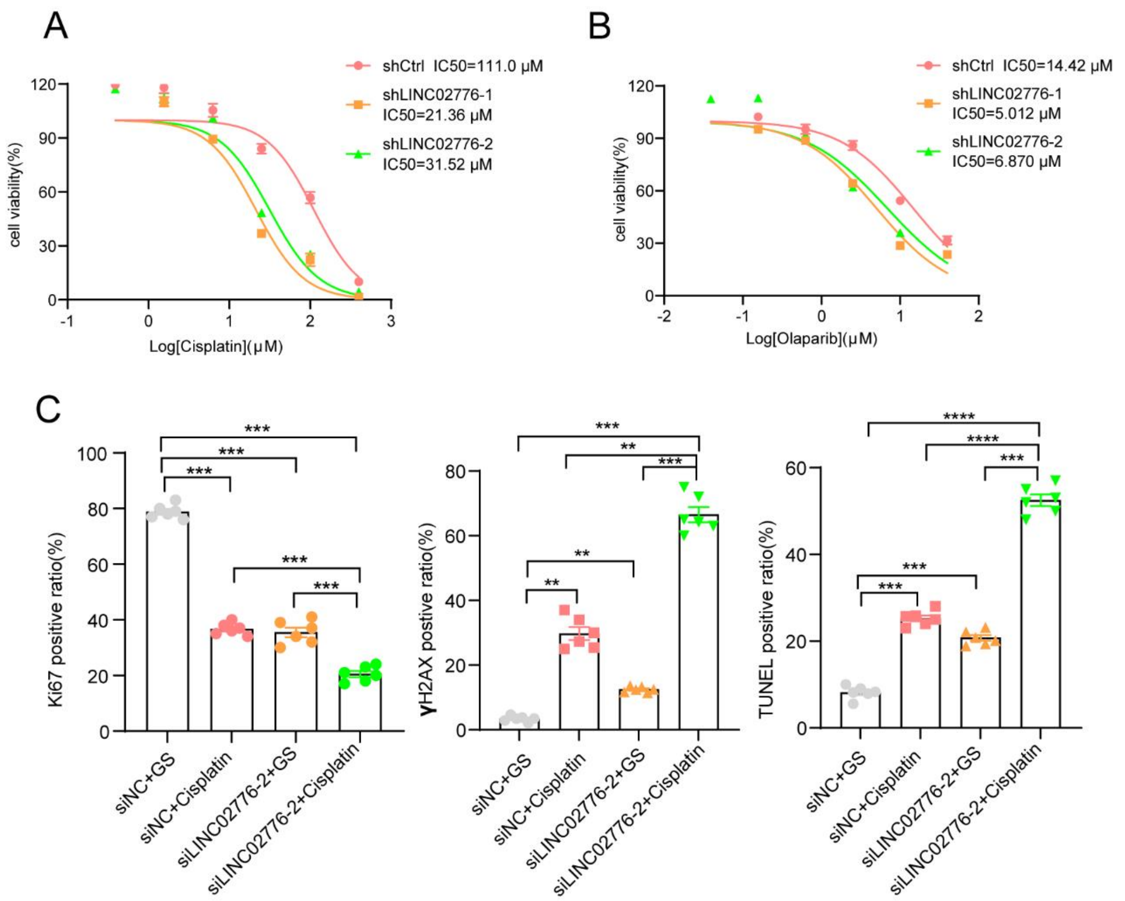

Supplementary Figure S11. LINC02776 knockdown enhances drug sensitivity in OC models. (**A**) CellTiter-Glo 3D assay results showing a significant reduction in cell viability in OC organoids following cisplatin treatment in the absence of LINC02776. (**B**) CellTiter-Glo 3D assay results demonstrating a significant decrease in cell viability in OC organoids after olaparib treatment when LINC02776 is knocked down. (**C**) Quantification of Ki-67-positive cells, γ-H2A.X-positive cells, and TUNEL-positive cells, corresponding to data presented in Figure 8F, evaluating cell proliferation, DNA damage, and apoptosis. Values are presented as mean ± SEM, n = 3 for panels (A) and (B). Statistical significance is indicated as follows: **P* < 0.05; ***P* < 0.01; ****P* < 0.001; *****P* < 0.0001.

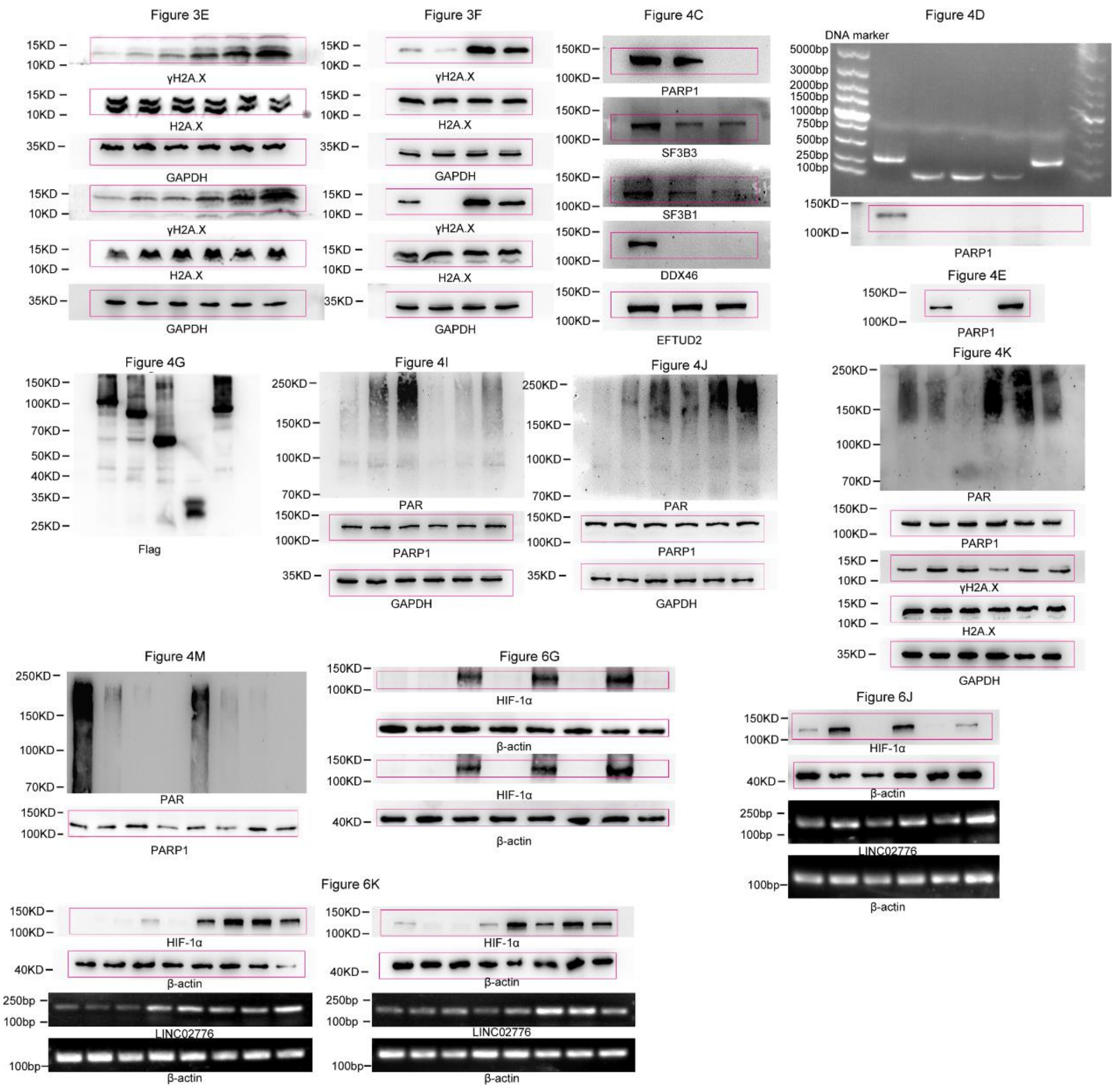

Supplementary Figure S12. Raw Western blot images of the corresponding figures.
